# Dual Chemical Looping/Catalytic Process for Alkylation of Benzene With Ethane and Propane Yielding Ethylbenzene and Cumene Over Copper‐Containing Mordenite

**DOI:** 10.1002/anie.202523668

**Published:** 2026-01-30

**Authors:** Florent J. Dubray, Yu‐Hsun Wang, Mikalai A. Artsiusheuski, Jiawei Guo, Rene Verel, Ambarish Kulkarni, Jeroen A. van Bokhoven, Vitaly L. Sushkevich

**Affiliations:** ^1^ Paul Scherrer Institute (PSI) Center For Energy and Environmental Sciences Villigen 5232 Switzerland; ^2^ Department of Chemical Engineering University of California Davis California 95616 USA; ^3^ Laboratory For the Science and Applications of Catalysis College of Chemistry University of California at Berkeley Berkeley California 94720 USA; ^4^ Institute For Chemical and Bioengineering ETH Zurich Vladimir‐Prelog‐Weg 1 Zurich 8093 Switzerland

**Keywords:** alkylation, chemical looping, copper‐containing zeolites, DFT calculations, NMR spectroscopy

## Abstract

Given the sustained demand for alkylated aromatics and the strained olefin market, there is an urgent need to develop efficient one‐step processes for the direct alkylation of aromatics using alkanes instead of olefins. Such technologies offer greater energy efficiency and sustainability by eliminating the need for separate, energy‐intensive alkane dehydrogenation steps. In this work, we report a dual chemical looping / catalytic process that couples alkane dehydrogenation with aromatic alkylation over a copper‐containing mordenite yielding up to 25% of alkylated aromatics with >97% selectivity per cycle. In situ MAS NMR and FTIR spectroscopies combined with DFT calculations showed that the alkylation of benzene with alkanes proceeds via a π‐bounded Cu(I)‐olefin intermediate, which subsequently interacts with benzene, catalyzed by Brønsted acid sites, leading to alkylated products that readily desorb from the active material into the gas phase. DFT calculations show that alkylation mediated solely by Cu(I) has prohibitively high barriers (>1.8 eV), whereas a bi‐functional pathway involving both Cu(I) and Brønsted acid sites can proceed with significantly lower barrier (0.8 eV) through a concerted C–C bond formation and proton transfer step.

## Graphical abstract

We report a dual chemical looping/catalytic process coupling alkane dehydrogenation with aromatic alkylation over Cu‐mordenite, yielding up to 25% alkylated aromatics with >95% selectivity per cycle. In situ NMR, FTIR spectroscopy, and DFT show alkylation proceeds via a π‐bound Cu(I)–olefin intermediate and Brønsted acid sites. DFT reveals a low‐barrier bifunctional pathway versus prohibitively high barriers for direct Cu(I)‐mediated mechanism.

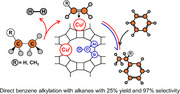

## 1 Introduction

Benzene is one of the most important aromatic compounds produced from oil refining, both in terms of quantity and diversity of use in today's industry. Most of the worldwide produced benzene is alkylated with light olefins to intermediate aromatic compounds that are used as basic materials for the production of solvents, surfactants, and plastics. Among those, approximately 70% of the benzene is alkylated either with ethylene or propylene to produce ethylbenzene and cumene, that are further involved in the industrial manufacturing of styrene, polystyrene, phenol, bisphenols, phenolic resins, and acetone among other polymers and specialty chemicals [1, 2]. The production of all those commodities is therefore dependent on the availability of light olefins, in particular ethylene and propylene.

Light olefins including ethylene and propylene sit at the cornerstone of the modern petrochemical industry. They are mostly produced as secondary products from fluid catalytic cracking (FCC) and shale gas and naphtha steam cracking, both of which are energy extensive processes with a large carbon dioxide footprint [3, 4, 5]. With the ever‐increasing demand on light olefins, a market‐shortage is therefore at place, urging the development and commercialization of on‐purpose olefin production technologies, such as non‐oxidative and oxidative dehydrogenation of alkanes [6, 7, 8, 9]. However, these processes suffer from significant environmental footprint, complex product separation, and either unfavorable thermodynamics at low temperatures (non‐oxidative path) or high selectivity to undesired oxidation products (oxidative path) [10, 11, 12, 13, 14, 15, 16, 17, 18, 19, 20, 21].

A direct use of alkanes in benzene alkylation excludes the need to produce olefins and potentially provides better process integration and efficiency. The possibility to react benzene with ethane was first demonstrated by Olah et al. using fluoroantimonic acid (HF‐SbF_5_) as a catalyst with a yield below 1 mol% [22]. Later on, benzene alkylation using ethane and propane was reported over bi‐functional heterogeneous catalysts, combining metal sites that are active in dehydrogenation reaction (such as Pt or Ga) and acidic zeolites [23, 24, 25, 26, 27, 28, 29, 30, 31, 32, 33, 34, 35, 36, 37]. These studies, however, revealed one of the main issues related to the direct alkylation of benzene with alkane, which is its complex selectivity control. Numerous side reactions significantly lower the overall process’ selectivity. Among others, (i) hydrogenolysis of alkanes and olefins, (ii) cracking, (iii) olefin oligomerization, and (iv) isomerization of alkylated aromatic products affect the process the most.

For the reaction of benzene with propane over Ga‐modified ZSM‐5, Bigey and Su reported high selectivity toward toluene and ethylbenzene, with cumene and *n‐*propylbenzene being minor products formed at relatively low temperature [30]. The authors suggested the predominance of high propane cracking rates over acid sites. This hypothesis was confirmed in the works by Ivanova et al [25]. and Derouane et al [37]. who demonstrated by ^13^C NMR that benzene alkylation with propane starts at around 573 K, with ethylbenzene and toluene being the major primary products. Such behavior was explained in terms of strong interaction of benzene with the catalyst at low temperature, which prevents the access of propane to the active sites. The reaction mechanism of direct benzene alkylation with propane over Pt‐MFI materials was further investigated by Smirnov et al., and strong effects of acid site density were demonstrated [31]. An increased Brønsted acidity resulted in higher propane conversion but lower selectivity to propylbenzenes due to the increased propane cracking and propylbenzenes dealkylation rates. The increase of Pt loading resulted in higher dehydrogenation of propane to propylene leading, in turn, to higher propane conversion and higher selectivity to propylbenzenes up to 44%. Further addition of a hydrogen scavenger (Zr_2_Fe) allowed to shift the thermodynamic equilibrium and resulted in higher conversions and selectivity to alkylated aromatics. The maximum yield of propylbenzenes reached 96% with respect to equilibrium, with a selectivity of 60%. In another work by Alotaibi et al., a heteropolyacid‐based catalyst was used for the benzene alkylation with propane, but benzene‐based conversion reached only 6%–8% with a cumene selectivity of 90%–93%.[34] It turns out that the first step of the mechanism of benzene alkylation with ethane over bi‐functional catalysts involves the dehydrogenation of ethane to ethylene over metallic sites, and then the subsequent benzene alkylation with ethylene over acid sites. The relatively low rate of ethane dehydrogenation typically results in low concentration of ethylene which prevents its side reactions as well as slows down the coking rates. Ethylbenzene selectivity of 92 to 95 mol% was achieved over Pt‐MFI catalyst, for a benzene‐based yield of 10%, but with an ethane to benzene ratio of 9:1, which means that the ethane‐based yield remains below 2% [32]. In work by Kato et al. a benzene‐based yield for ethylbenzene of 7.3% was achieved over Pt modified ZSM‐5 and ZSM‐22 zeolites. However, styrene formation (yield 0.4%) was also observed, highlighting that use of such catalytic systems can lead to secondary reactions of alkylated products [23].

The one‐step direct alkylation of benzene with alkanes inherently depends on first dehydrogenating the alkane to form an olefin, followed by acid‐catalyzed alkylation. However, the dehydrogenation step is thermodynamically unfavorable and typically demands high temperatures above 673 K. In contrast, the optimal temperature for acid‐catalyzed alkylation is around 523 K. At elevated temperatures, undesirable side reactions such as C–C bond cleavage and olefin oligomerization become prominent [25, 37, 38, 39, 40]. The ultimate need for high catalytic activity of both active sites under the same reaction conditions typically leads to the initiation of numerous side‐reactions and fast coking, as described above. As a result, low benzene‐based yields with even lower alkane‐based yields are generally observed, associated with relatively poor selectivity toward alkylated aromatic compounds.

Copper‐containing zeolites were already shown to possess catalytic activity in alkylation of benzene with methanol formed via chemical looping oxidation of methane [41]. Recently, we have developed a novel chemical looping strategy for the dehydrogenation of light alkanes to olefins over copper(I) centers hosted in mordenite (MOR) zeolite [42, 43]. The formation of stable olefin‐copper(I) π‐complexes uniquely allows achieving high olefin yields and selectivity at low temperature. *A priori*, the stability of such π‐complexes limits the contribution of the side reactions and potentially enables to use them as alkylating agents at the favorably low temperature. In the present work we, for the first time, demonstrate the selective benzene alkylation with alkanes via dual chemical looping / catalytic process using copper(I)‐containing zeolite. Using in situ FTIR and MAS NMR spectroscopies supported by DFT calculations we elucidate the key steps of the reaction mechanism. As a first step, alkane reacts with Cu(I) mordenite to produce a strong π‐bounded Cu(I)‐olefin complex and gaseous hydrogen. At the second step, benzene interaction with the Cu(I)–olefin complex is catalyzed by the neighboring Brønsted acid sites, where the concerted C–C bond formation and proton transfer step take place yielding alkylated products with high selectivity. We demonstrate that the dual chemical looping/catalytic process over copper(I)‐containing zeolites enables the selective direct one‐step conversion of ethane and propane and benzene into high‐value ethylbenzene and cumene hence offering a new low‐temperature route for the valorization of alkanes.

## 2 Results and Discussion

**FIGURE 1:**
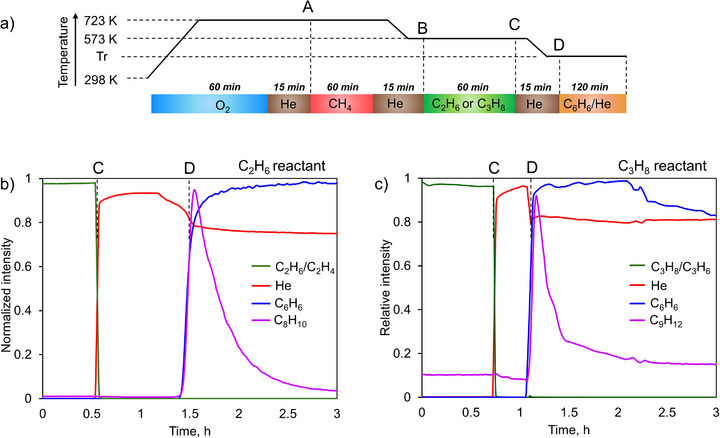
(a) A scheme describing the chemical looping / catalytic alkylation process. The A, B, C, and D marks correspond to changes in a gas flow and temperature (described in more detail in the Supporting Information). A is the starting point of material reduction with methane, B and C are the start and the end of the alkane dehydrogenation step, respectively, D is the start of the benzene alkylation step. (b) and (c) show the normalized MS signals during the alkylation step for ethane and propane, respectively, both conducted at 573 K, plotted against time on stream. Each MS signal is normalized to its maximal intensity over the experiment.

The copper‐containing mordenite Cu(II)‐MOR material used in this work was prepared by an ion exchange method. The mordenite Si/Al ratio was 6.5, copper loading was 4.35 wt%, corresponding to 690 µmol·g^−1^. The XRD pattern (Figure S1) and nitrogen physisorption analysis (Figure S2) of the Cu(II)‐MOR material as well as detailed Cu(II)‐MOR synthesis procedure are available in the Supporting Information. Cu(I)‐MOR was prepared from Cu(II)‐MOR by reduction in methane prior to further experiments [43]. In the first step of the chemical looping protocol, the alkane was always reacted for 1 h at 573 K with the freshly prepared Cu(I)‐MOR sample (Figure 1a). The amount of formed olefin during 1 h reaction was separately quantified by water‐assisted desorption at 348 K using on‐line micro‐GC, preliminary calibrated with standard mixtures. A total of 220 µmol·g^−1^ of ethylene and 310 µmol·g^−1^ of propylene were formed (Figure S3). This corresponds to the conversion of approximately 32% and 45% of the total copper(I) sites available in the Cu(I)‐MOR for ethane and propane reactants, respectively. This is in line with our previous work, where 1 h reaction at 573 K resulted in fractional formation of π‐bounded olefin complexes [42, 43]. To keep an equal olefin content bound on Cu(I)‐MOR for each alkylation tests, the dehydrogenation reaction conditions were fixed as described above.

**FIGURE 2:**
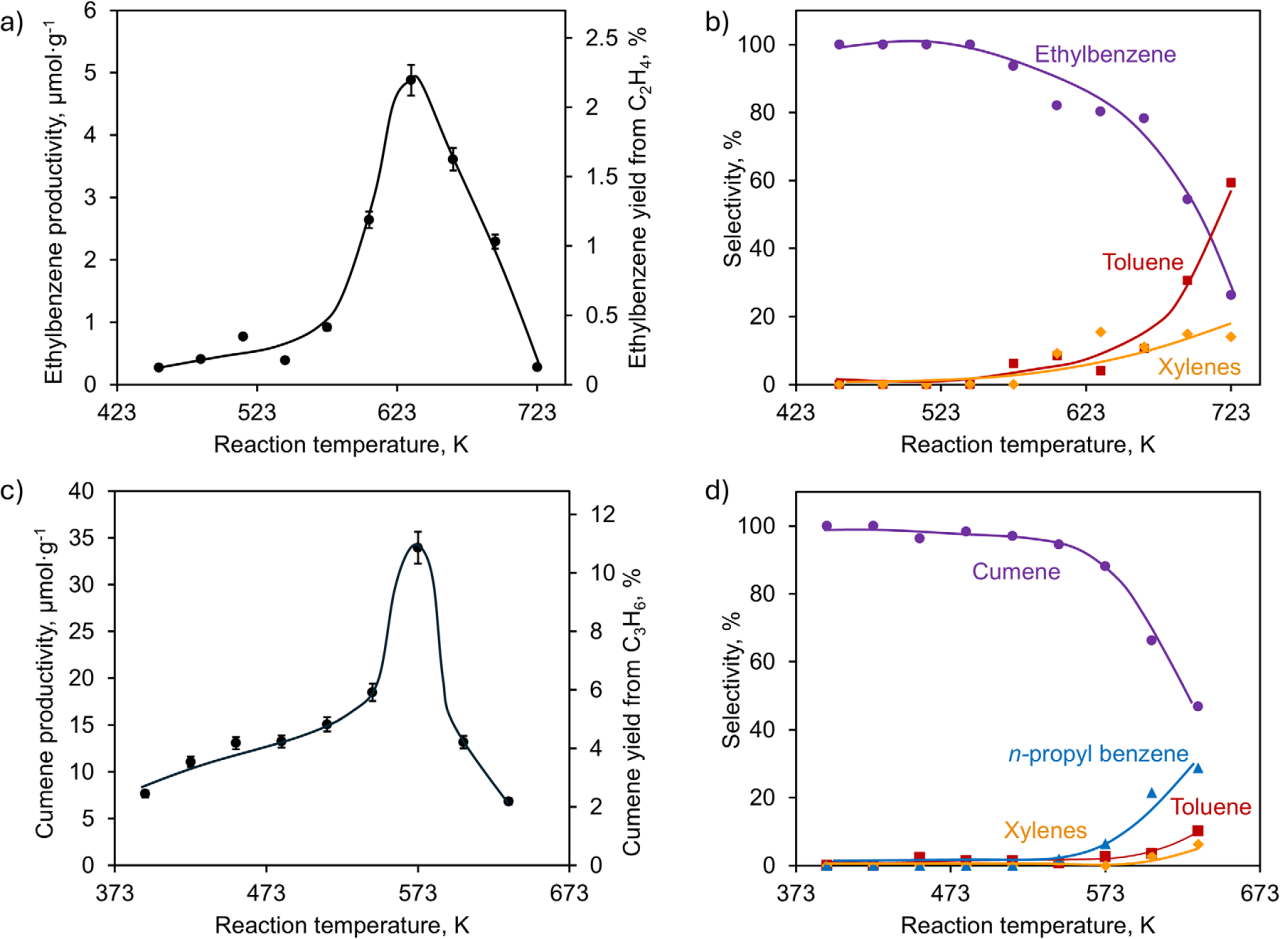
Ethylbenzene productivity and yield (a) and selectivity (b) measured at different reaction temperatures. Similarly, the cumene production and yield (c) and selectivity (d) versus reaction temperature. Solid lines are to guide the eye.

The reactivity of the π‐bounded olefins toward benzene was then evaluated by reacting them with a benzene‐containing stream at 573 K while monitoring the evolution of products with a mass spectrometer directly connected to the reactor outlet (Figure 1b,c). Upon contact between benzene vapors and Cu(I)‐MOR containing π‐bounded ethylene respectively propylene, the alkylation products, predominantly ethylbenzene and cumene, respectively, were immediately detected in the gas phase: the MS signal for alkylated products sharply rises to its maximum value followed by progressive decrease until the reaction is complete after around 2 h time on stream. Interestingly, no alkanes or olefins were detected in the gas phase, indicating that all olefins undergo reaction with benzene, possibly due to the high excess of benzene in the feed, in line with spectroscopic and DFT results (vide infra).

To quantitatively study the reactivity of π‐bounded olefins for the benzene alkylation reaction, the MS analyzer was replaced with a dichloromethane trap where the reaction products were collected and analyzed with conventional GC‐FID using 1,4‐dioxane as the external standard. Figures 2 and S4 show the effect of temperature on the yield and selectivity of the reaction. In the case of ethane, alkylation with benzene resulted in the formation of predominantly ethylbenzene. The maximal yield toward ethylbenzene of 4.9 µmol·g^−1^ was achieved at 633 K, corresponding to 2.3 mol% of available alkene. Below 523 K, the ethylbenzene selectivity of almost 100 % is reached while at higher temperature toluene and xylene were also observed, suggesting the occurrence of cracking and transalkylation side reactions. Propane alkylation with benzene resulted in the predominant formation of cumene. Similar to ethane, below 523 K, the selectivity toward cumene is close to 100 %, but increasing the reaction temperature to above 573 K resulted in the formation of toluene, xylenes, ethylbenzene, and *n*‐propylbenzene as by‐products. Among them, *n*‐propylbenzene was the most substantial, showing selectivity up to 30%. Surprisingly, the yield of cumene remained relatively low, with maximum of 34 µmol·g^−1^ or 11.0 mol% based on the available π‐bounded propylene. This value is higher than that obtained for ethane, but still below the theoretical maximum. Aside from the reactivity associated with olefins, additional formation of phenylbenzene and naphthalene was observed at all reaction temperatures and attributed to the dehydrogenative coupling of benzene over the metal and acid sites in Cu(I)‐MOR [44]. A repetition test conducted at 573 K (Figure S5) showed a slow decrease of cumene yield after each subsequent regeneration, with an activity loss after 5 cycles of about 43%, although the deactivation remained relatively low (below 15%) for the first three cycles.

**FIGURE 3:**
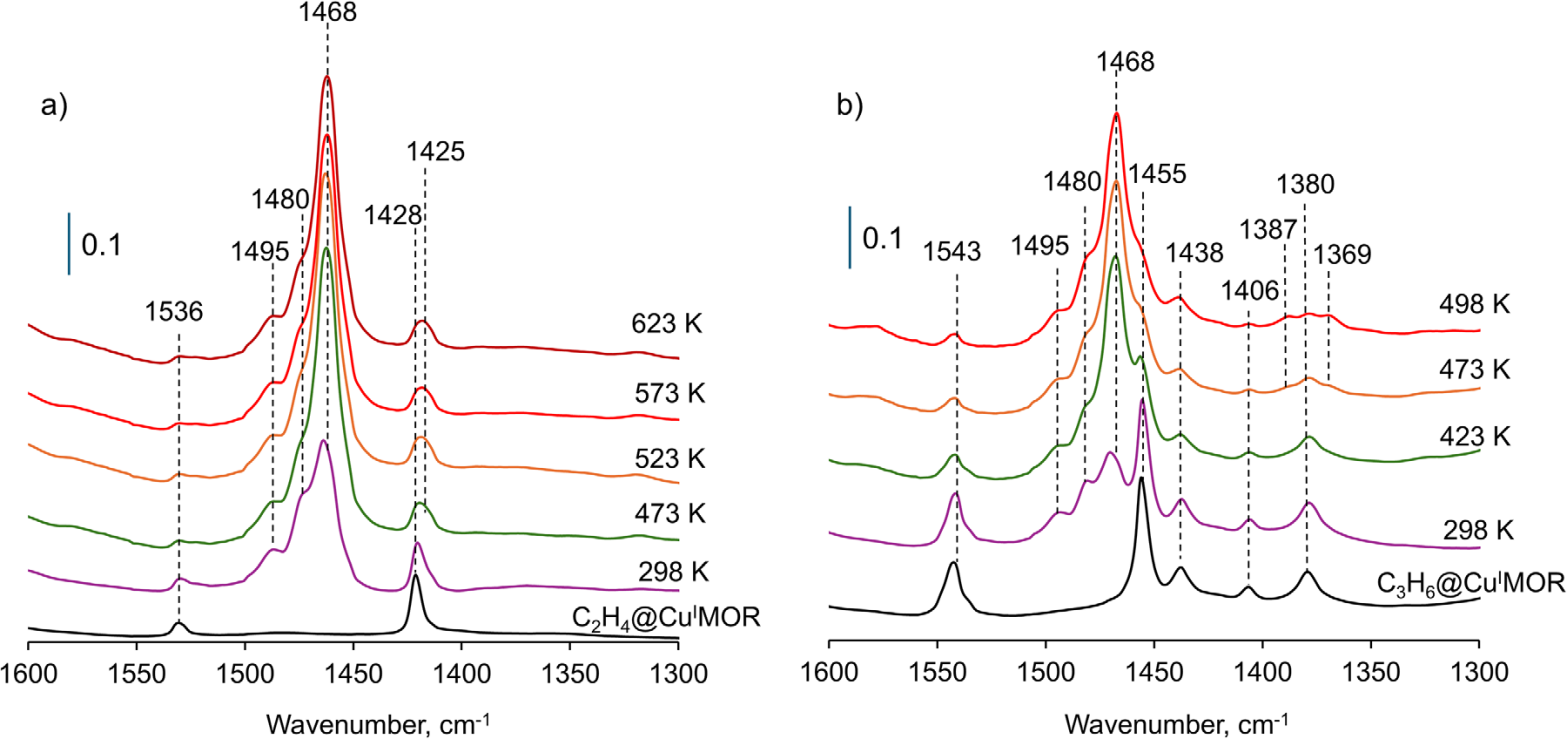
In situ FTIR difference spectra of (a) ethane‐reacted Cu(I)‐MOR and ethane‐reacted Cu(I)‐MOR exposed to benzene at different temperatures, and (b) propane‐loaded Cu(I)‐MOR and propane‐loaded Cu(I)‐MOR exposed to benzene at different temperatures. Reference spectrum for the difference is the activated Cu(I)‐MOR. The data presented in this figure also available as difference spectra (Figure S7), further highlighting the consumption of FTIR bands related to olefins products, and the formation of bands associated with ethylbenzene and cumene.

The reactivity of π‐bounded olefins for the benzene alkylation was also investigated using situ FTIR spectroscopy to assess possible reaction intermediates. Figure 3a displays that π‐bounded ethylene has vibration bands at 1536 and 1428 cm^−1^ ascribed to the C═C stretching vibrations and ═CH_2_ scissoring vibrations modes of ethylene molecule, respectively [42, 45, 46, 47]. The addition of benzene at ambient conditions resulted in the appearance of new IR bands located at 1495, 1480, and 1468 cm^−1^, that are all attributed to benzene vibration modes [48]. At ambient temperature and after benzene was introduced, the IR bands ascribed to π‐bounded ethylene vibration modes remain unchanged. This demonstrates that at ambient temperature, benzene is not able to trigger the desorption of π‐bounded ethylene, and no benzene alkylation is taking place. Upon increasing the temperature to above 473 K, the signals of IR bands ascribed to π‐bounded ethylene are diminishing, suggesting the consumption of the olefin species. Notably, no bands associated with gas phase ethylene are observed, but instead, a new IR signal corresponding to surface species was observed at approximately 1425 cm^−1^. This finding suggests that the π‐bounded ethylene is directly reacting with benzene, enabling its alkylation, without desorption into the gas phase.

In the case of propylene (Figure 3b), the bands at 1543 and 1455 cm^−1^ are due to the C═C stretching, and ═CH_2_ scissoring vibrations modes of Cu(I) π‐bounded propylene, respectively. Additional IR bands were also observed at 1438, 1406, and 1380 cm^−1^ and assigned to C─H deformation vibrations [42, 49, 50, 51]. Similar to the case of ethane, the addition of benzene at ambient temperature leads to the appearance of the bands at 1495, 1480, and 1468 cm^−1^ with no changes in the bands due to π‐bounded propylene. With increasing the reaction temperature to above 423 K, the intensity of bands associated with propylene was diminished and the development of new bands located at 1387 and 1369 cm^−1^ was observed and ascribed to C─H deformation modes of cumene [52]. No bands that could be associated with gaseous propylene were detected. These observations suggest that, similarly to ethylene, propylene π‐bounded species are inactive toward reaction with benzene at ambient temperature, but they are directly involved in the benzene alkylation above 423 K, without any evidence of olefins desorption into the gaseous phase, in line with the MS data obtained in the reactor tests (Figure 1). Notably, the reaction of benzene with π‐bounded propylene is initiated at lower temperature compared to the reaction with ethylene.

Adsorption of benzene was performed to clarify the assignment of the FTIR bands at 1495, 1480, and 1468 cm^−1^. In particular, an in situ freshly‐prepared Cu(I)‐MOR sample was exposed to benzene at increasing partial pressure‐ followed by acquisition of FTIR spectra at ambient temperature (Figure S6). At low benzene pressures, two major bands at 1468 and 1480 cm^−1^ are visible, possibly due to the interaction with copper (I) sites, while the introduction of significant excess of benzene leads to the appearance of the band at 1495 cm^−1^ due to weakly bound benzene.

**FIGURE 4:**
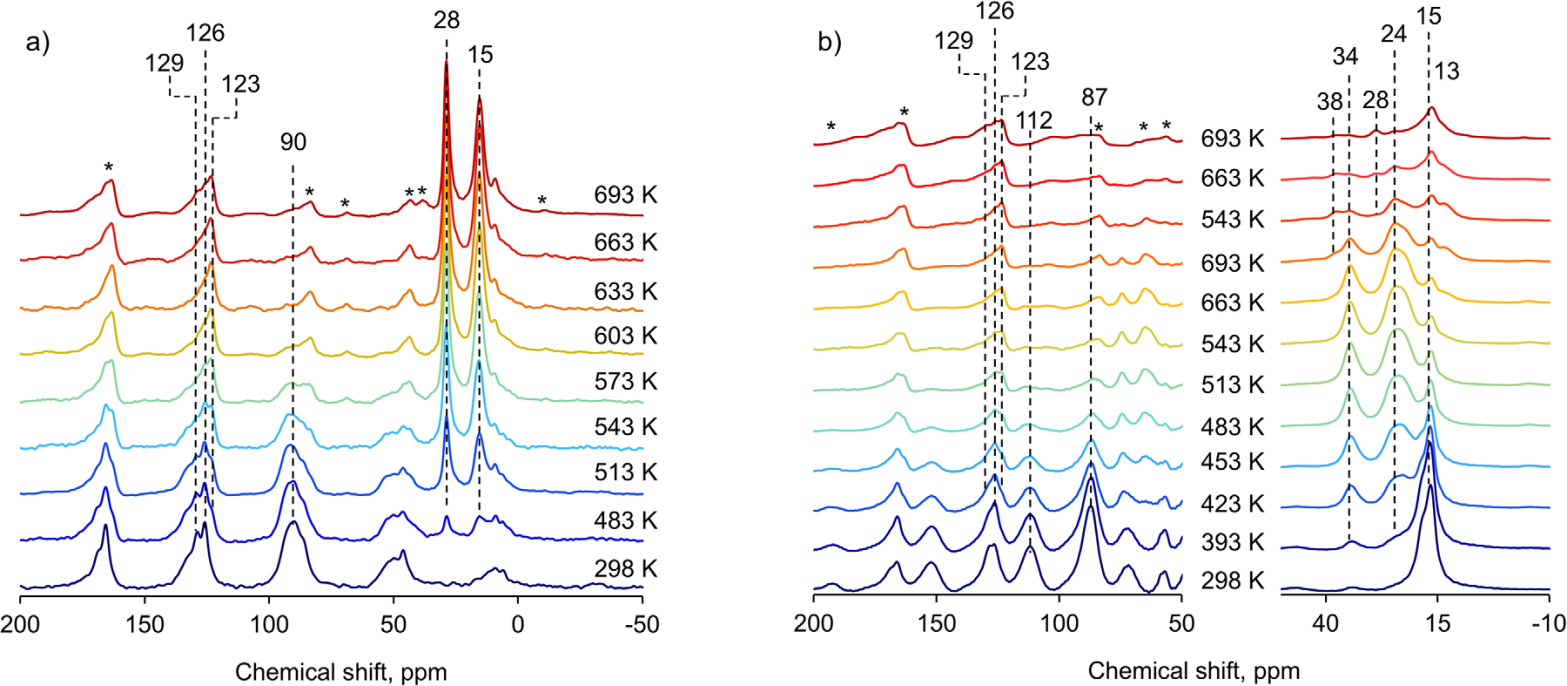
In situ (a) ^1^H–^13^C CP MAS NMR spectra acquired during the reaction of ethane‐reacted Cu(I)‐MOR with benzene at different temperatures. Similarly, (b) represent in situ ^1^H–^13^C CP MAS NMR spectra acquired during the reacting propane‐reacted Cu(I)‐MOR with benzene. Asterisks designate spinning side bands.

To further assess the mechanism of alkylation, the reaction was studied by means of in situ magic angle spinning (MAS) NMR spectroscopy. Figure 4a shows the in situ ^1^H–^13^C cross‐polarization (CP) MAS NMR spectra acquired during the reaction of π‐bonded ethylene with benzene over Cu(I)‐MOR. At ambient temperature, no reaction occurs, and the spectrum contains an intense signal at 90 ppm accompanied by spinning side band pattern attributed to the ethylene π‐bounded to Cu(I) sites [42]. Additionally, two signals located at 126 and 129 ppm are associated with benzene adsorbed in the zeolite pores and in the gas phase, respectively [25, 37, 53]. Upon increasing the reaction temperature, the intensity of the signal due to π‐bounded ethylene gradually decreases and new intense signals at 15 and 28 ppm appear and gradually develop. These are ascribed to ─CH_3_ and ─CH_2_‐ groups of ethylbenzene, formed upon reaction of ethylene with benzene [25, 37, 53]. In addition, the evolution of convoluted signals in the aromatic region and appearance of the shoulder at 123 ppm suggests the formation of new aromatic species, specifically ethylbenzene. Importantly, the intensity of these signals did not change significantly, pointing to the absence of isotope scrambling and/or ethylene desorption or aromatization. At 603 K, the ethylene signal at 90 ppm entirely disappeared, and the signals associated with the ethyl group in ethylbenzene achieved their maximum in intensity, suggesting that the alkylation reaction was complete at this stage. No evidence for any gaseous phase olefin species was observed in direct excitation ^13^C spectra (Figure S9), in agreement with FTIR data and reactor tests (see Supplementary information). The measurements using a MAS spinning speed of 3 and 4 kHz (Figure S8) excluded possible signal overlap with spinning side bands, hence confirming the original assignments.

Figure 4b shows ^1^H–^13^C CP MAS NMR spectra obtained during the reaction of propylene with benzene over Cu(I)‐MOR. The presence of π‐bounded propylene was evidenced by two signals at 87 and 112 ppm due to ═CH_2_ and ═CH─ carbon atoms together with a sharp signal at 16 ppm attributed to the ─CH_3_ group [42]. Upon increasing the reaction temperature to above 423 K, new signals appeared at 24 and 34 ppm, respectively attributed to the ─CH_3_ and ─CH─ groups in cumene [25, 37, 53]. This also came along with an evolution of signals in the aromatic region (123–129 ppm), suggesting the apparition of new aromatic species, similarly to the case of Cu(I) π‐bounded ethylene reactions with benzene. In parallel to the formation of cumene, the intensity of the signals at 112, 87, and 16 ppm gradually decreased, pointing to the consumption of π‐bounded propylene species. When the reaction temperature of 483 K was reached, no signals related to π‐bounded propylene or to gaseous phase propylene (expected at 135, 115, and 19 ppm respectively for ═CH─, ═CH_2_, and ─CH_3_) were observed, and the cumene signals at 24 and 34 ppm ceased to grow. This suggests that full conversion of the π‐bounded propylene species to alkylated products was achieved. At reaction temperatures above 573 K, new signals started to emerge at 13 and 34 ppm respectively, attributed to ─CH_3_ and ─CH_2_─ carbons from *n*‐propylbenzene, together with a shift of the signal at around 24 ppm inferred to the additional contribution of the ─CH_2_─ carbon from *n*‐propylbenzene [25, 37, 53]. The observation of *n*‐propylbenzene as alkylated products is in line with the reactor test, where selectivity toward *n*‐propylbenzene increases with reaction temperature. Further increase of the reaction temperature to above 573 K resulted in a weak signal at 28 ppm, corresponding to CH_2_ group of ethylbenzene. An additional signal at ‐4 ppm was attributed to methane, pointing to the cracking of cumene and/or propylbenzene that may be responsible for the formation of ethylbenzene, as discussed above. [25, 37] In addition, in situ ^1^H MAS NMR and direct excitation ^13^C with high‐power proton decoupling (HPDEC) spectra for the reaction of ethane and propane reacted Cu(I)‐MOR with benzene at different temperatures (Figures S9 and S10) confirmed complete conversion of π‐bounded olefins. The reaction with propane occurs at a lower temperature than that for π‐bounded ethylene (Figures S9 and S10), in line with FTIR data and reactor tests. This is translated to the highest yield toward alkylated products being reached at ∼633 K from ethane, and at ∼573 K from propane, indicating a higher reactivity of Cu(I) π‐bounded propylene for the benzene alkylation reaction. ^1^H MAS NMR spectra show the presence of the signal at around ∼ 4 ppm due to Brønsted acid sites (BAS) at all stages of the reaction for both ethylene and propylene alkylation (Figures S9 and S11). A slight variation in the intensity versus reaction temperature might be associated with the severe broadening of the peak due to interaction with reagents and reaction products and hence, ambiguity in integration.

Building on these spectroscopic and reactor test results, the next step was to elucidate possible reaction pathways and active‐site requirements. We employed periodic density functional theory (DFT) calculations to probe the elementary steps and assess the feasibility of different mechanistic scenarios. Details of our DFT calculations, performed with the RPBE functional with Grimme's D3(BJ) corrections using the Vienna ab‐initio simulation package, are provided in the Supporting Information.[54, 55, 56] We consider several possible active sites and reaction mechanisms to rationalize the above experimental observations. Specifically, we investigated a direct alkylation route on a Cu(I) site and a bi‐functional pathway that requires both the Cu(I) site and a Brønsted acid site in the same unit cell. For the bi‐functional pathway, the impact of having one or two benzene molecules in the zeolite pore was taken into account. Our data shows (Figure S12) that direct interaction of benzene with ethylene π‐bonded to Cu(I) has a free energy barrier of ∼1.8 eV at 298 K that increases to 2.0 eV at 623 K. As the experiments show that ethylbenzene forms at temperatures lower than 623 K, we conclude that direct mechanism is most probably not in place. Next, we considered a pathway over bi‐functional active site consisting of a Brønsted acid site (BAS) located near the Cu(I) cation (Figure S13), which implied the ethylene desorption, formation of framework‐bound ethoxy species, and C─C bond formation to yield ethylbenzene. Although the energetics of this mechanism are more favorable than the direct pathway outlined above (Figures S12 and S13), we note that our FTIR and NMR experimental data do not show formation of framework bound ethoxy species as well as gas phase ethylene. Thus, we consider another mechanism that sidesteps the formation of a framework bound ethoxy intermediate.

**FIGURE 5:**
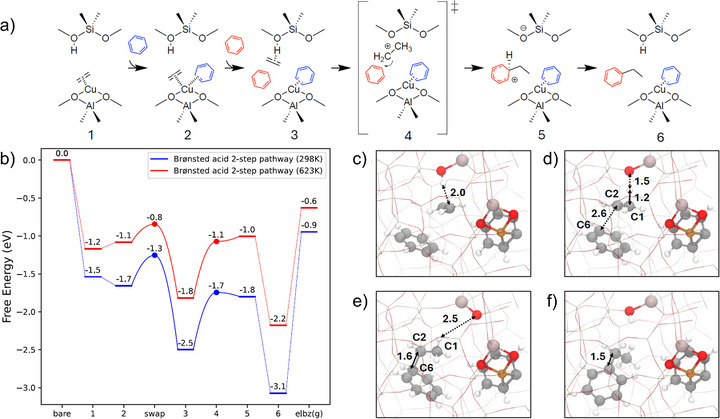
(a) Proposed 2‐step reaction scheme for benzene alkylation reaction mediated by the BAS/Cu site with the concerted path. (b) Corresponding free energy profile calculated at 298 K (blue) and 623 K (red). (c–f) Optimized structures of the key intermediates and transition state along the pathway, corresponding to states 3–6 in panel (a). Color scheme: C (gray), H (white), O (red), Cu (brown), Al (pink). Numbers in subfigures c)–f) correspond to distance in Å.

Specifically, using the same active site (i.e., Cu(I)/BAS) as above, we explore if and how addition of a second benzene molecule changes the mechanistic picture (Figure 5a). The motivation for this comes from the experimental conditions implying the utilization of large excess of benzene during the alkylation. Analogous to the 3‐step pathway discussed above, here, the first benzene molecule (denoted in blue) facilitates ethylene desorption. But, instead of forming an ethoxy intermediate by reacting with the BAS as discussed in 3‐step mechanism (Figure S13), the desorbed ethylene reacts with a second benzene molecule (shown in red, Figure 5a) to form a protonated ethylbenzene. The final step is its deprotonation, which yields the ethylbenzene product and regenerates the Brønsted acid site. We denote this pathway as the 2‐step mechanism as the formation of ethoxy species intermediates (i.e., Figure S13d–f) does not take place. Figure 5b displays the free energy diagram corresponding to this scheme together with the geometries of the key intermediates summarized in Figures 5c–f. The energetics of the initial steps (1–3) remain similar to the ones in Figure S13b but ethylbenzene formation occurs via a concerted mechanism rather than a two‐step process. The key branching point is the fate of the desorbed ethylene that is physiosorbed on the Brønsted acid site (Figures 5c and S13d). Instead of forming a stable framework ethoxy species, we observe a transition state (Figure 5d) where the hydrogen atom transfer from the Brønsted acid site to C1_Et_ occurs simultaneously with C─C bond formation between C2_Et_ and C6_Bz_. This concerted, bi‐functional pathway, which leads to the formation of a protonated ethylbenzene, has a barrier of 0.8 eV at 298 K; this barrier decreases to 0.7 eV at 623 K. These values are consistent with previous calculations by Xing et al.[57] The final deprotonation step is energetically favorable and leads to the final ethylbenzene product. As the calculated barriers for this bi‐functional concerted pathway are lower than the other two mechanisms considered above, and it agrees with absence of gas‐phase olefins or formation of ethoxy species, we propose this as most plausible mechanism of the three we attempted.

**FIGURE 6:**
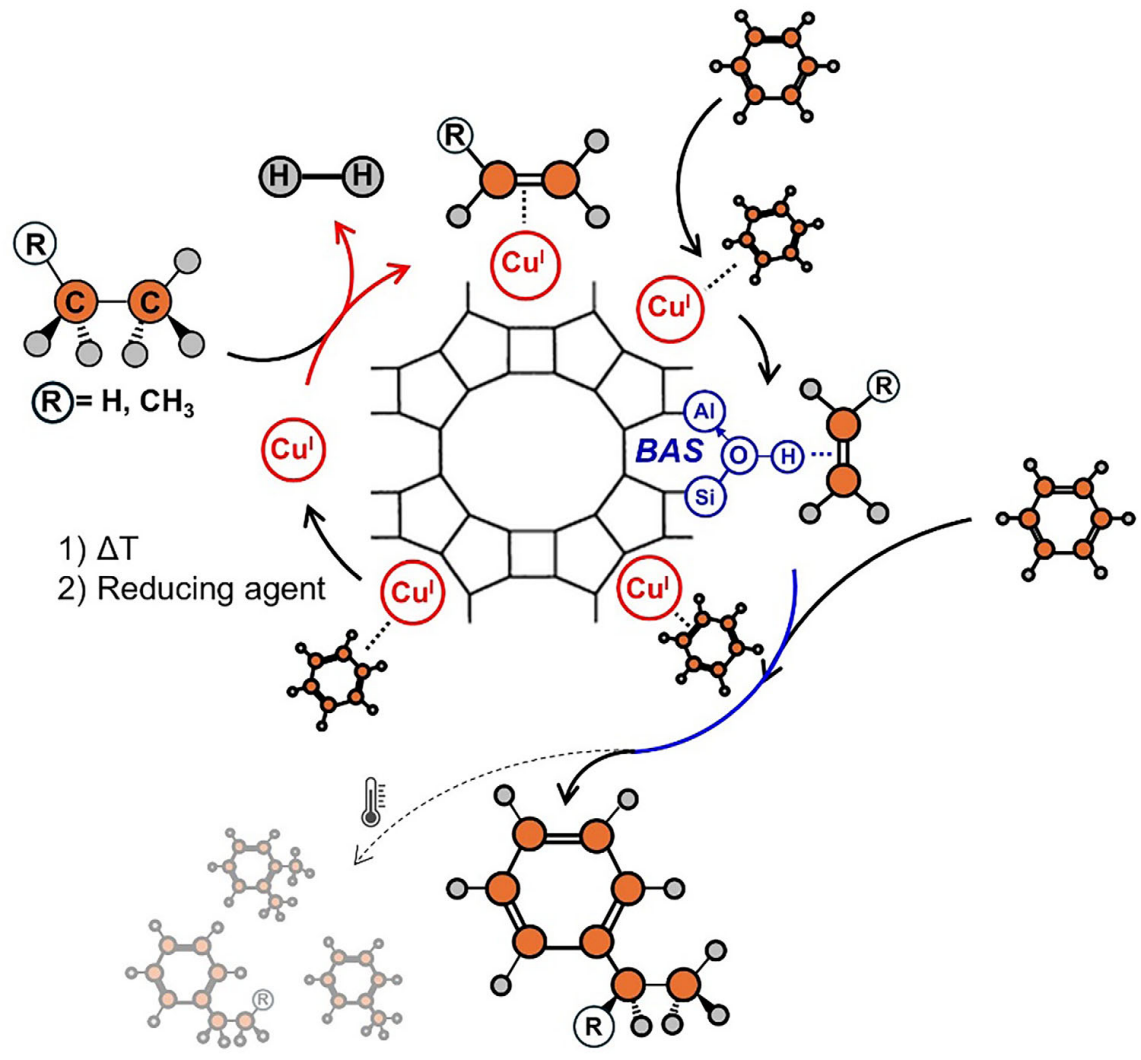
Scheme of the dual chemical looping/catalytic alkylation of benzene with alkane (ethane or propane) over Cu(I)‐MOR.

Figure 6 summarizes the reactions taking place during direct benzene alkylation with ethane and propane on Cu(I)‐MOR. When alkanes are reacted with copper(I) sites in Cu(I)‐MOR, they are dehydrogenated and formed alkenes remained π‐bounded to the Cu(I) site, while hydrogen is liberated into the gas phase. Once these π‐bounded olefins species are contacted with benzene at ∼400–500 K, alkylation of the benzene occurs resulting in the consumption of the Cu(I)‐olefin complexes, and the formation of alkylated aromatic products, namely, ethylbenzene and cumene from π‐bounded ethylene and propylene, respectively. Cumene is formed preferentially over *n*‐propylbenzene as evidenced by the high cumene selectivity at low temperatures (below 573 K). At high temperatures, side reactions such as non‐selective alkylation, cracking, trans‐alkylation, and isomerization occur being responsible for the loss of selectivity toward ethylbenzene and cumene. Cracking was further evidenced by the formation of methane.

In situ NMR spectroscopy confirmed the complete conversion of alkenes and absence of other by‐products. Therefore, the low amount of ethylbenzene and cumene extracted in the reactor tests might be associated with diffusion limitations. Indeed, we evidenced the presence of trace amount of phenylbenzene and naphthalene as side products from dehydrogenative condensation/coupling of benzene over Cu(I)‐MOR. The resulting polyaromatic compounds are bulky molecules which can block the pores of the MOR structure hence trapping alkylation reaction products inside the zeolite pores. This is further confirmed by the fact that in the reactor test, significant excess of benzene is flown through the catalyst enabling the formation and possible accumulation of polyaromatic compounds. To further assess this hypothesis, a mesoporous Cu(I)‐MOR_m_ sample was prepared and tested for the propane to cumene reaction at 573 K. Despite a lower copper content of 1.12 wt%, the resulting cumene yield accounted to 25%. This supports the idea that relatively low yields of alkylated products are retrieved from the reactor tests at least partially because of diffusion limitation leading to secondary reactions, thus opening a way for further process improvement through the choice of nano‐sized, mesoporous, or hierarchical zeolites structures, and/or zeolite with larger pores.

To study the effect of different Cu/BAS ratio to the reaction, we prepared the copper‐exchanged MOR sample, using ammonium form of mordenite, which activation leads to higher number of BAS produced by decomposition of NH_4_^+^ counter‐cation. The resulting activated sample designated at “HCu(I)‐MOR” showed the presence of more BAS as evidenced from the FTIR spectra in the region of bridging OH groups (Figure S14s and S15). We estimated the number of BAS in the newly prepared sample as 750 µmol·g^−1^ versus 500 µmol·g^−1^ in the original sample prepared by using Na‐form of mordenite. Utilization of this sample prepared using NH_4_‐form of mordenite and possessing 50% more of Brønsted acid sites (750 µmol g^−1^, designated as HCu(I)‐MOR) resulted in improved catalytic activity. In the reactor test performed at 573 K, 43 µmol g^−1^ of cumene was formed (vs 34 µmol·g^−1^ for Cu(I)‐MOR) with the selectivity of 90 %. This highlights the importance of BAS for the overall process and provides the direction for further optimization of catalyst composition.

## 3 Conclusions

The direct alkylation of benzene with ethane and propane to ethylbenzene and cumene was achieved in a dual chemical looping / catalytic process using copper(I)‐containing mordenite. FTIR and MAS NMR spectroscopy data demonstrated that the reaction proceeds in two steps: first, dehydrogenation of alkanes at Cu(I) sites to yield stable π‐bonded Cu(I)‐olefin complexes and molecular hydrogen; second, alkylation of benzene with these intermediates at neighboring Brønsted acid sites, affording alkylated products with high selectivity at low temperatures. Complete conversion of olefins can be achieved, while the desorption of the alkylation products into the gas phase is hindered. Equally, the use of mesoporous zeolite allows to significantly improve the cumulative yield of alkylated aromatic compounds. Further optimization of the reaction process may also involve the introduction of small amount of water vapors, which might facilitate the desorption of olefins, making them readily available for alkylation reaction. DFT calculations show that a bifunctional mechanism involving both Cu(I) sites and Brønsted acid sites is identified as the most plausible route, proceeding through a concerted C–C bond formation and proton transfer step with a significantly lower barrier (∼0.8 eV). This combined experimental and computational evidence highlights the cooperative role of Cu(I)–olefin complexes and Brønsted acidity in enabling selective one‐step alkylation of aromatics with alkanes.

## Conflicts of Interest

The authors declare no conflict of interest.

## Supporting information




**Supporting File 1**: anie71322‐sup‐0001‐SuppMat.docx.

## Data Availability

The data that support the findings of this study are available from the corresponding author upon reasonable request.
