# Supplementary material for: Dual Chemical Looping/Catalytic Process for Alkylation of Benzene With Ethane and Propane Yielding Ethylbenzene and Cumene Over Copper‐Containing Mordenite
Source: Angew Chem Int Ed Engl. 2026 Jan 30;65(10):e23668. doi: 10.1002/anie.202523668 (PMC12955518; doi:10.1002/anie.202523668)
Supplement: Supplementary file 1 — Supporting File 1: anie71322‐sup‐0001‐SuppMat.docx. [file ANIE-65-e23668-s001.docx]

Supporting Information for

**Dual Chemical Looping/Catalytic Process for Alkylation of Benzene with Ethane and Propane Yielding Ethylbenzene and Cumene over Copper-Containing Mordenite**

Florent J. Dubray^†^, Yu-Hsun Wang ^˧^, Mikalai A. Artsiusheuski^†,§^, Jiawei Guo ^˧^, Rene Verel*^‡^*, Ambarish Kulkarni^*,˧^, Jeroen A. van Bokhoven^*,†,‡^, Vitaly L. Sushkevich^*,†^

*† Paul Scherrer Institute (PSI), Center for Energy and Environmental Sciences, 5232, Villigen, Switzerland*

*˧ Department of Chemical Engineering, University of California, Davis, California 95616, United States*

§ *Laboratory for the Science and Applications of Catalysis, College of Chemistry, University of California at Berkeley, CA, USA, 94720*

*‡ Institute for Chemical and Bioengineering, ETH Zurich, Vladimir-Prelog-Weg 1, Zurich 8093, Switzerland*

** Corresponding Author: Tel.: +41563103518; email:* [*arkulkarni@ucdavis.edu*](mailto:arkulkarni@ucdavis.edu)*,* [*jeroen.vanbokhoven@chem.ethz.ch*](mailto:jeroen.vanbokhoven@chem.ethz.ch)*;* [*vitaly.sushkevich@psi.ch*](mailto:vitaly.sushkevich@psi.ch)

**Experimental Section**

*Zeolite synthesis*

The Cu(II)-MOR material was prepared by ion exchange of a sodium-containing commercial mordenite purchased from Zeolyst (CBV10A) with a Si/Al ratio of 6. In a typical synthesis, ion exchange is performed by contacting for 12 h at 323 K the sodium mordenite (5 g) with 500 mL of a solution at 0.05 M of copper (II) nitrate (99+%, Sigma-Aldrich). The solid are then separated through filtration on a paper porous filter, rinsed with deionized water and dried at 393 K overnight. The procedure is repeated for a total of three times.

The dry sample was then pressed into a self-supported wafer, crushed and sieved with mesh size 0.25 - 0.5 mm. The as-obtained catalyst is denoted “Cu(II)-MOR”, and has a copper loading of 690 µmol.g^-1^ (4.4 wt% ; Si/Al = 6.5 ; Cu/Al = 0.38 ; Na content = 1.2 wt%) as determined by ICP-MS elemental analysis. The corresponding recorded PXRD pattern and N_2_-sorption data of the Cu(I)-MOR material are shown in Figures S1 and S2, respectively.

Mesoporous mordenite for Cu(II)-MOR_m_ sample was prepared by acid leaching of parent CBV10A. 10 grams of zeolite were treated in 0.5 M solution of HNO_3_ at 323 K overnight. The final powder was filtered, rinsed with deionized water and dried at 373 K. It underwent same ion exchange procedure with copper nitrate solution. 1.12 wt% of copper were introduced. N_2_-sorption data indicated the formation of 0.08 cm^3^·g^-1^ over mesopores with 0.20 cm^3^·g^-1^ of micropores.

To study the effect of different Cu/BAS ratio to the reaction, we prepared the copper-exchanged MOR sample, using ammonium form of mordenite, which activation leads to higher number of BAS produced by decomposition of NH_4_^+^ counter-cation. Ammonium form of mordenite (CBV10A) was prepared by triple ion exchange of 5 g of NaMOR in 500 ml of 0.1 M solution of NH_4_NO_3_. The final material was filtered, rinsed with deionized water, and dried at 393K overnight. The number of BAS cannot be directly measured using FTIR spectroscopy of adsorbed pyridine due too small 8 MR side pockets of MOR structure, inaccessible to pyridine. We used an indirect approach to estimate the number of BAS in the Cu(I)-MOR. We separately prepared a fully exchanged NH_4_-form of parent MOR sample by triple ion exchange of sodium form in the solution of NH_4_NO_3_. This sample was in situ calcined under vacuum in the IR setup into HMOR and the total intensity of the band due to bridging OH groups was measured. Then, we made an assumption, that all aluminum measured by ICP-OES is forming Bronsted acid sites, and no Lewis acid sites is formed, since we did not observe any significant bands at around 3660 cm^-1^ due to Al-OH groups associated with extra-framework species. Then, integration of the 3608 cm^-1^ FTIR band corresponding to Brønsted acid sites, in the activated Cu(I)-MOR resulted in approximately ~500 µmol/g (Figure S14 and S15).

The copper-containing mordenite with increased number of Brønsted acid sites, designated as “HCu(II)-MOR” was prepared using the resulting ammonium form of CBV10A. 4 g of NH_4_-form of mordenite was ion exchanged in 400 ml of 0.05 M aqueous solution of copper nitrate at 333K overnight. The resulting material was filtered, rinsed with deionized water, and dried at 393K. The procedure was repeated three times. The conversion of “HCu(II)-MOR” into “HCu(I)-MOR” possessing reduced copper (I) sites was performed identically to Cu(I)-MOR sample by treating in reducing agent at high temperature, see below.

The resulting activated sample designated at “HCu(I)-MOR” showed the presence of more BAS as evidenced from the FTIR spectra in the region of bridging OH groups (Figure S14 and S15). We estimated the number of BAS in the newly prepared sample as 750 µmol/g versus 500 µmol/g in the original sample prepared by using Na-form of mordenite.

*Analysis*

^1^H and ^13^C MAS NMR spectra were recorded on an AVANCE IIIHD 400WB (Bruker BioSpin) spectrometer having a field of 9.4 T, equipped with a double-resonance 7 mm MAS probe (Bruker BioSpin). The magic angle spinning frequency was set to 4 kHz. The high power proton decoupling (HPDEC) ^13^C spectra were acquired using a direct excitation on the carbon channel associated with a high-power proton decoupling during the acquisition with a TPPM decoupling pulse sequence, and a recycling delay of 2 s.^1^ The cross-polarization ^1^H-^13^C CP MAS NMR spectra was acquired with a modified high-power proton decoupling, namely the CP-RAMP modification with TPPM, still with a recycle delay of 2 s, and a contact time of 1.5 ms.^1^ The ^13^C chemical shift was calibrated using an adamantane external reference (38.6 ppm). The free induction decays (FID) were all zero-filled and apodized through the multiplication of a decaying exponential function associated to a line broadening of 32 Hz. Fourier-transform was then applied, and phase was manually adjusted. Signal intensities were normalized to the number of scans, and data processing was performed on TopSpin 3.6 software.

Fourier transformed infrared spectra (FTIR) were measured using an iS50 spectrometer equipped with a DTGC detector (Thermo Scientific). Spectra were collected in transmission mode with an optical resolution of ~4 cm^-1^ and by averaging 128 scans. To obtain solely the spectra of surface species, the reference spectrum was subtracted from all spectra, with the reference spectrum being measured right prior to alkane reactive dehydrogenating.

Powdered X-Ray diffraction (XRD) patterns were recorded with a Bruker D8 diffractometer using the Cu Kα1 radiation (λ = 1.5456 Å). Nitrogen sorption was acquired on a 3-flex Micromeritics unit. The sample is first activated in vacuum at 573 K before being subjected to N_2_ sorption experiments (equilibration time = 10 s).

Samples collected from reaction tests were primarily analyzed with gas chromatography equipped with an FID detector (GC-FID), collected on a 6890 Series Agilent Technologies gas chromatograph equipped with a 122-5032 DB-5 capillary column (30 m, 250 µm diameter, 0.25 µm wall thickness). Injection port temperature was set to 493 K and the column was subjected to the following thermal treatment: 5.5 min at 308 K, then heating-up with a 10 K/min ramp up to 588 K. Helium is used as the carrier gas. Attribution of signal was performed through the use of reference chemicals that were measured so as to know their corresponding retention time, and further confirmed using another gas chromatograph equipped with a mass spectrometer detector (GC-MS: 7890A Series Agilent Technology gas chromatograph equipped with a 5975C inert XL MSD with triple-axis detector mass spectrometer). The measurement conditions for the GC-MS were kept the same. Amount of olefin formed from alkanes on the Cu(I)-MOR was quantified through water-assisted desorption (as described hereinafter in reactor-test section), using a micro-GC equipped with a MS5 molecular sieve and using Argon as carrier gas (time of analysis = 2 min, injection temperature = 373 K, column temperature = 393 K, column pressure = 140 kPa). Quantification was achieved through external calibration (for micro-GC and GC-FID signals). In the case of GC-FID measurements from reaction tests, the reactions products are dissolved in an unknown amount of dichloromethane. Quantification was achieved through the quantified addition of 1,4-dioxane standards to the solutions (1,4-dioxane being used as a reference for quantification).

Yields (for reactor tests) were calculated based on Equation (1). For ethylbenzene formation, the corresponding olefin is ethylene, while for cumene formation, propylene is used instead. Ethylene and propylene were monitored and quantified using micro-GC in water desorption tests.

$Yield\left( alkylated aromatic \right)=\frac{n(alkylated aromatic)}{n(\pi-bounded olefin)}$ (1)

Selectivity was calculated based on reaction products involved with π-bounded olefin transformation (therefore excluding benzene, naphthalene and phenylbenzene), such that the selectivity of reaction product x (x and i being different from benzene, phenylbenzene and naphthalene) can be written according to Equation (2).

$Selectivity\left( x \right)=\frac{n(x)}{\sum_{i} n(i)}$ (2)

For MAS NMR experiments, conversion of π-bounded ethylene was evaluated as the relative consumption of the HPDEC ^13^C NMR corresponding signal located at 90 ppm. In the case of propylene, the corresponding signal located at 87 ppm was used instead. Consumption was evaluated by taking the sample measured at ambient conditions as the reference.

*Chemical looping process for reactor test*

In a typical reactor test, about 150 mg of dried and shaped Cu(II)-MOR is introduced in a stainless-steel tubular reactor (6.35 mm in diameter) and maintained in the middle of the reactor with glass wool supported on a quartz rod. The Cu(I)-MOR is then activated under pure O_2_ atmosphere (28 ccm, with ccm standing for normal cubic centimeters per minute) at 723 K for 1 h, to burn off any organic impurity and remove water. The O_2_ flow is then switched to He flow (40 ccm) for 15 minutes so that all O_2_ is purged. The He flow is then replaced by a methane (Messer, 5.0) flow (50 ccm) for 1 h, with the temperature still at 723 K, so that the copper species from the Cu(II)-MOR are reduced to Cu(I) species (step A in Figure 1a). The temperature is then decreased to the dehydrogenation temperature of 573 K, still under methane flow. The methane flow is then turned off and replaced by the alkene of choice between ethane (Carbagas, 4.5, 34 ccm) or propane (Carbagas, 3.5, 25 ccm), for exactly 1h (step B in Figure 1a). The flow of alkane is then replaced with helium (40 ccm) to remove any unreacted gas phase alkane from the reactor (step C in Figure 1a). The temperature is then adjusted to the desired reaction temperature Tr, still under He flow. Once the temperature is stabilized, the alkylation step is performed by diminishing the He flow down to 30 ccm, and re-routing it through a bubbler placed at room temperature filled with pure benzene (Merck, 99.5%), so that benzene vapors are flown into the reactor, triggering the reaction with the Cu(I) π-bounded olefins (step D in Figure 1a) followed by the desorption into the gas phase. Simultaneously, the gas-flow exiting the reactor is re-routed towards a bubbler filled with dichloromethane (CH_2_Cl_2,_ Merck, 99.5%) which purpose is to absorb all reaction products exiting the reactor for analysis. The reactive desorption is conducted for a total of 2 h. Then, the bubblers are disconnected from the set-up, and He is flown at 40 ccm for 15 min to evacuate all remaining gas from the reactor.

The content of the dichloromethane-containing bubbler is then mixed with a known amount of 1,4-dioxane (Merck, 99.5%) used as an external standard, and injected in GC-FID.

In case of re-cycling, the flow of the reactor is switched to O_2_ (28 ccm), and the overall procedure can be repeated. One repetition of the procedure accounts for what is called one chemical looping cycle.

For reaction test performed with mass spectrometry (MS) analysis, the dichloromethane bubbler was not attached; instead, the reactor outlet was directly connected to a MS detector. The signals at m/z = 4, 28, 30, 78, 92, 106, 29, 41, and 105 were followed, and respectively associated to helium, ethylene, ethane, benzene, ethylbenzene, propane, propylene, and cumene.

For quantification of the total olefin content that is π-bounded to the Cu(I)-MOR zeolite, experiments where benzene is replaced with pure water are conducted (with a desorption temperature Tr fixed at 348 K to avoid any side reaction such as isomerization or oligomerization). In such case, the dichloromethane bubbler is not installed, and replaced by a connection to a micro-GC gas analyzer, for quantification of the olefin being desorbed.

*In situ FTIR spectroscopy*

Static reaction tests are performed in a batch equivalent of the reactor flow procedure. For IR in-situ analysis, the Cu(II)-MOR in powdered form (approximately 20 mg) is shaped as a self-supported wafer (~2 cm^2^) and pressed with a hydraulic press at 0.5 metric ton. The sample is then introduced in a custom-made quartz-cell equipped with a furnace.

The Cu(II)-MOR is first subjected to vacuum, then 300 Torr of O_2_ is added and maintained in static conditions while the pellet is activated at 723 K for 1 h (8 K.min^-1^ ramp). Then, the Cu(II)-MOR is subjected to vacuum for 1 h to evacuate any combustion products (including water), with the Cu(II)-MOR being maintained at 723 K, with a residual pressure of ~3.10^-6^ Torr. Reduction of the Cu(II)-MOR into Cu(I) species is then conducted by inserting 300 Torr of pure CO gas (still at 723 K) and keeping the entire system under static conditions for 1 h. The CO is then evacuated with vacuum that is applied for a duration of 1 h (still at 723 K) to a residual pressure of ~3.10^-6^ Torr. The Cu(I)-MOR is then kept under vacuum while temperature is brought to 573 K. The corresponding alkane (ethane or propane) is then introduced (300 Torr) and contacted for 1 h under static conditions. Vacuum is then pulled for 1 h, and temperature ramped down to 298 K. Then, 5 equivalents of benzene are introduced as a vapor (equivalents are calculated as a function of the total olefin amount that is π-bounded to the zeolite, as evaluated with the reactor run where water was used to desorb the olefin).

The IR cell is then isolated and IR spectra is recorded. Then, the pellet is heated up to reaction temperature Tr for 5 min, and placed back at 298 K for spectra acquisition.

*In situ NMR spectroscopy*

For NMR in-situ analysis, the raw unshaped powder (~80 mg) is used and inserted into a 5.59 mm outer-diameter and ~1.0 cm working length glass tube.

Cu(I)-MOR was prepared using a procedure identical to described above in the in situ FTIR experiments. For the alkane adsorption, either ^13^C_1_-ethane or ^13^C_3_-propane (99%, Sigma Aldrich) was introduced (300 Torr) and contacted for 1 h under static conditions at 573 K with the freshly reduced Cu(I)-MOR sample. Vacuum is then pulled for 1 h, and temperature is ramped down to 298 K. Then, 5 equivalents of benzene (unlabeled, with natural ^13^C abundance) are introduced as a vapor (equivalents are calculated as a function of the total olefin amount that is π-bounded to the zeolite, as evaluated from the reactor run where water was used to desorb the olefin from the Cu(I)-MOR material). The resulting overall system is then cooled down using liquid nitrogen and the glass tube containing the Cu(I)-MOR loaded with alkane and benzene is sealed-off, being kept at 77 K (using an open flame). The sealed sample is then inserted into a 7 mm diameter zirconium rotor, so as to be measured in MAS-NMR. The glass ampoule is then successively heated up to various reaction temperatures Tr (in increasing order of temperature) in a static oven for 5 min, while NMR data are collected in-between each temperature, under ambient conditions. Spinning bands were unambiguously assigned with the help of a spectra recorded at a different MAS rotation speed of 3 kHz.


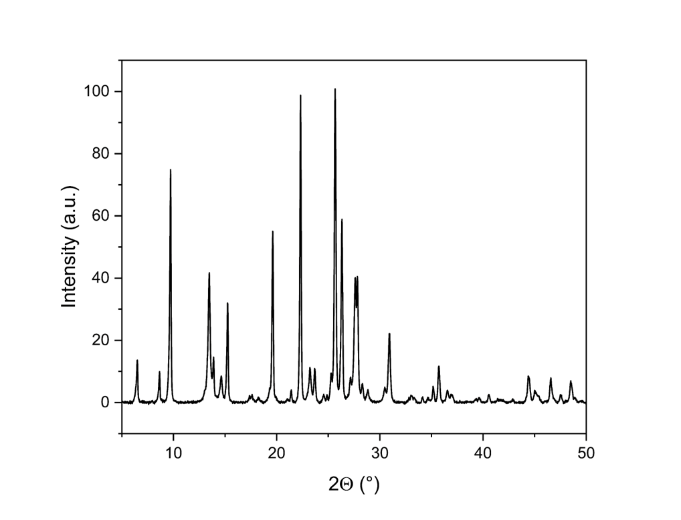


**Figure S1**: PXRD pattern for Cu(I)-MOR material


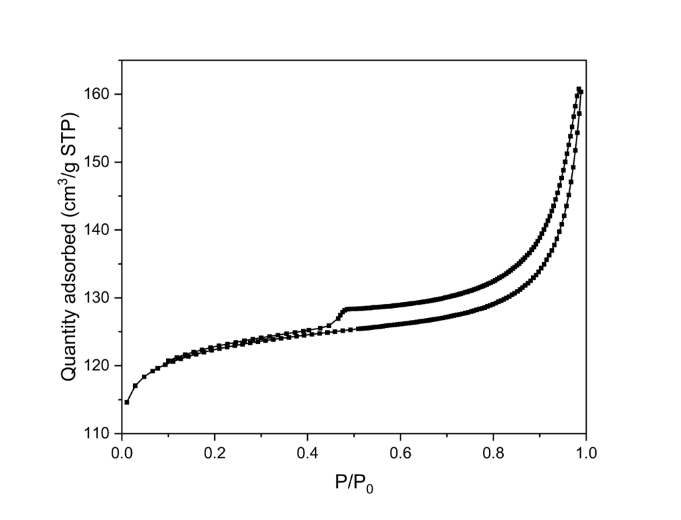


**Figure S2**: N_2_-sorption isotherm (showing adsorption and desorption hysteresis) of Cu(I)-MOR material


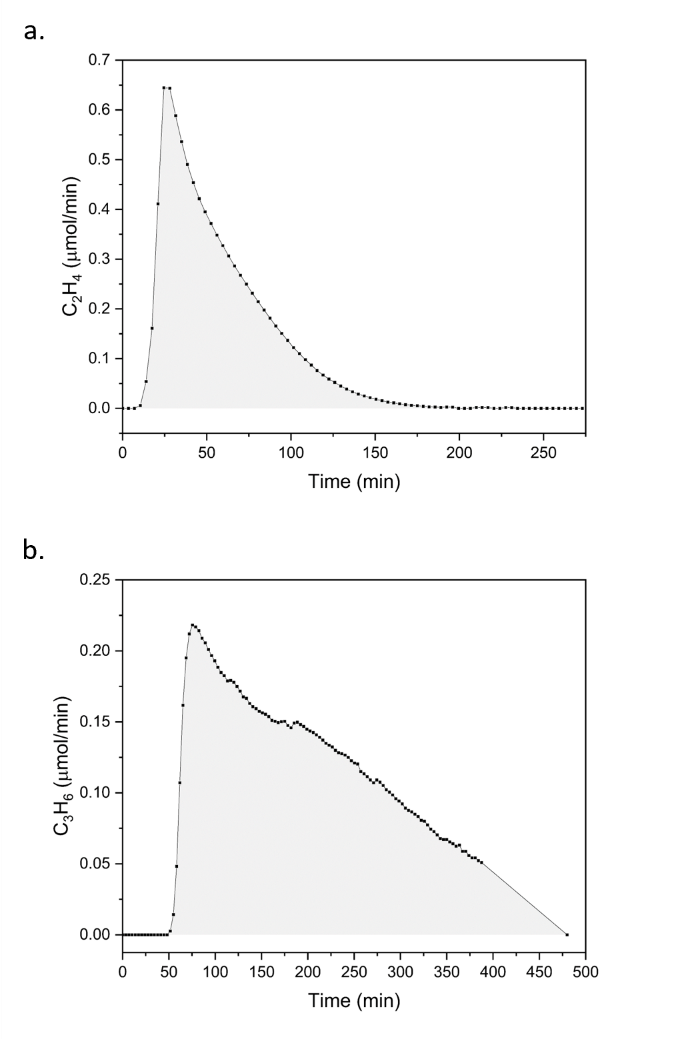


**Figure S3**: Desorption of (a) ethylene and (b) propylene loaded Cu(I)-MOR with water at 348 K, tracked with the corresponding micro-GC signal for ethylene and propylene respectively, used for the quantification of the total olefin content that is π-bounded to the Cu(I)-MOR support. Desorption time = 0 is set arbitrarily, slightly before desorption is started for clarity.


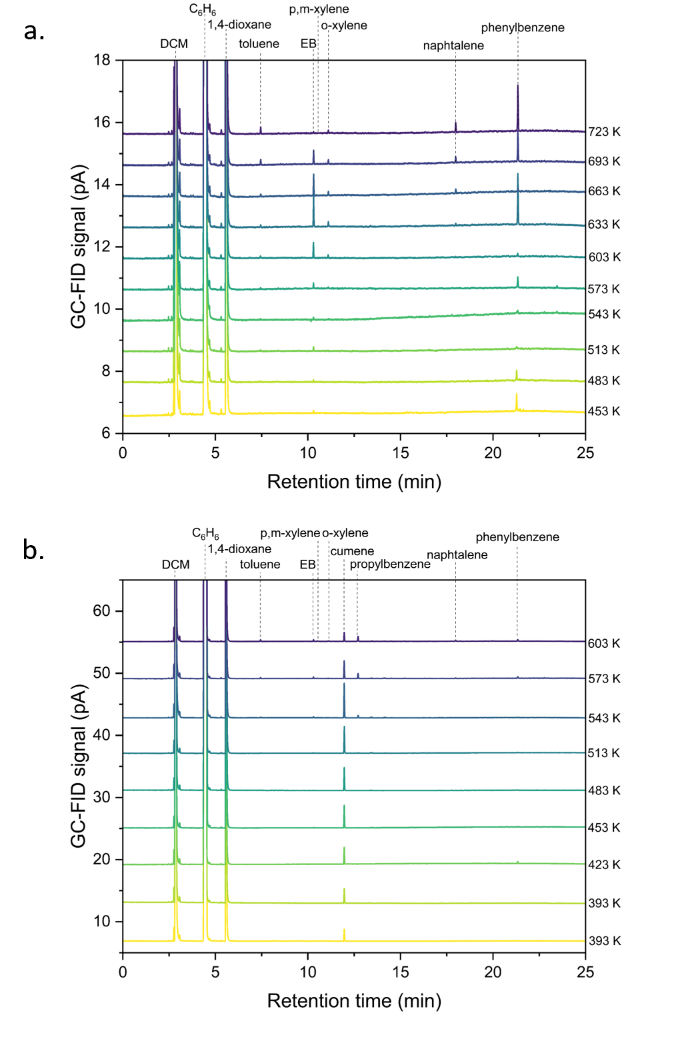


**Figure S4**: Raw GC-FID signal recorded for reactor tests at various reaction temperature Tr for (a) ethane to ethylbenzene alkylation reaction and (b) propane to cumene alkylation reaction. Signals attribution is highlighted.


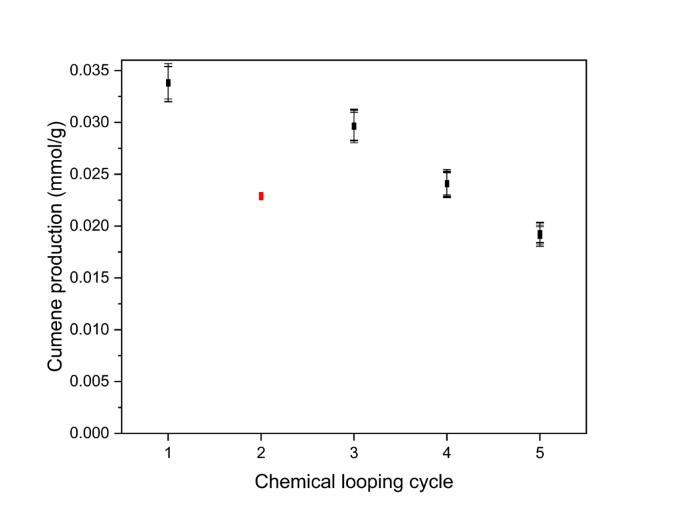


**Figure S5**: Repetition test of propane to cumene alkylation performed at 573 K, for five successive times. Cycle #2 is an outlier due to improper quantification of the corresponding GC-FID signal.


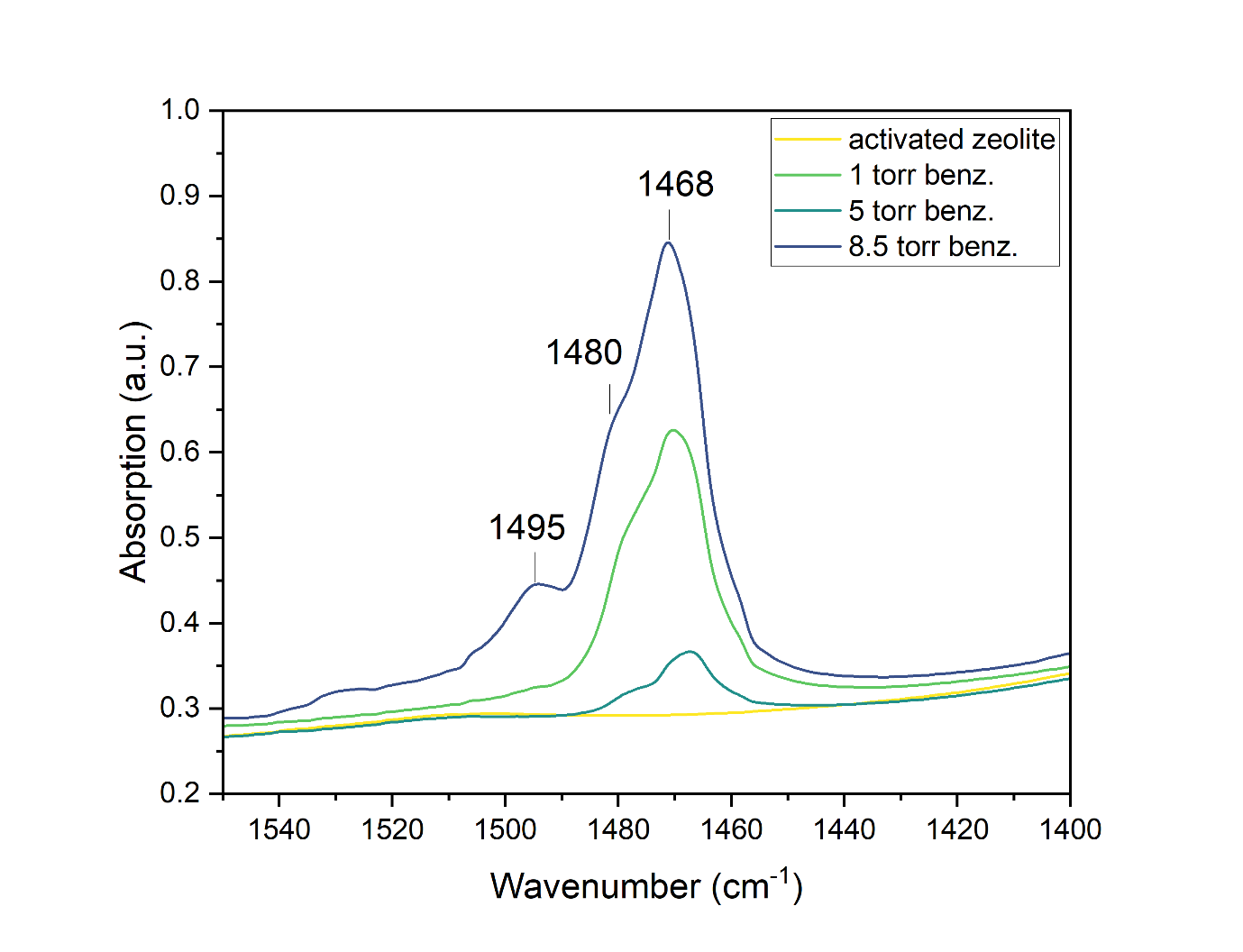


**Figure S6:** FTIR spectra of benzene adsorption over the activated Cu(I)-MOR sample.


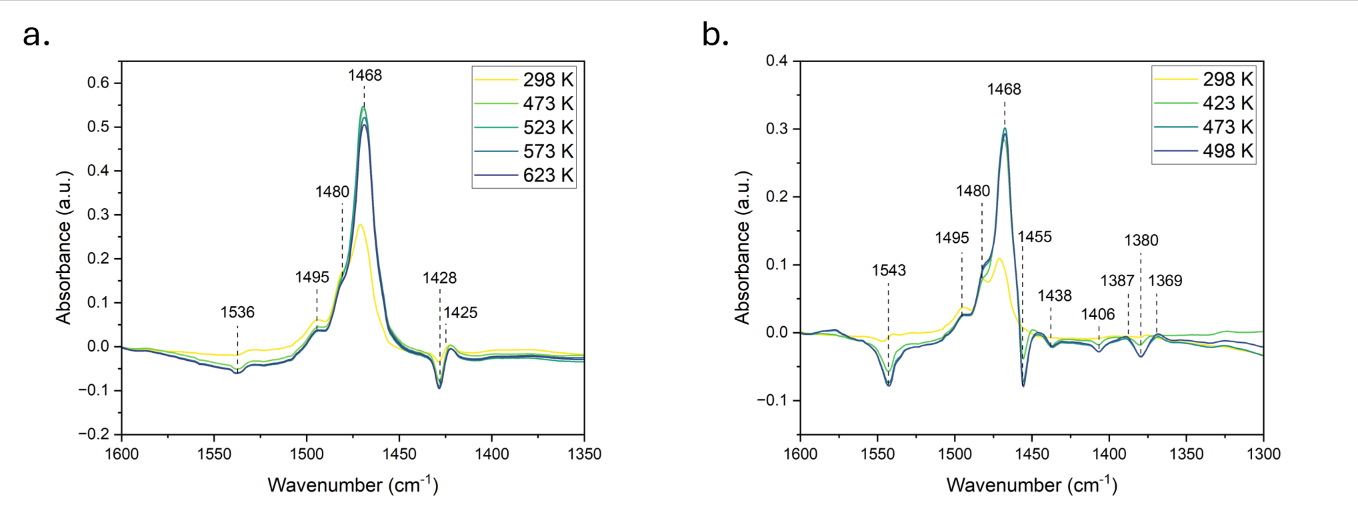


**Figure S7**: FTIR difference spectra obtained by subtracting the spectrum with (a) formed ethylene or (b) formed propylene taken prior benzene introduction, from the other samples taken at increasing reaction temperatures.


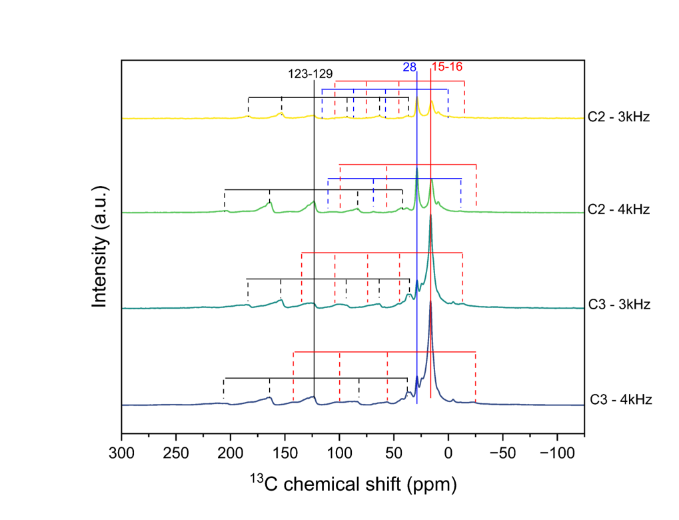


**Figure S8**: ^1^H-^13^C CP MAS NMR of ethylene to ethylbenzene (denoted “C2”) and propylene to cumene (denoted “C3”), measured after reaction at 693 K at two different MAS rotation speed: 3 kHz and 4 kHz. Plain lines are direct signals, and in dotted lines are indicated their corresponding spinning side bands, with the corresponding color.


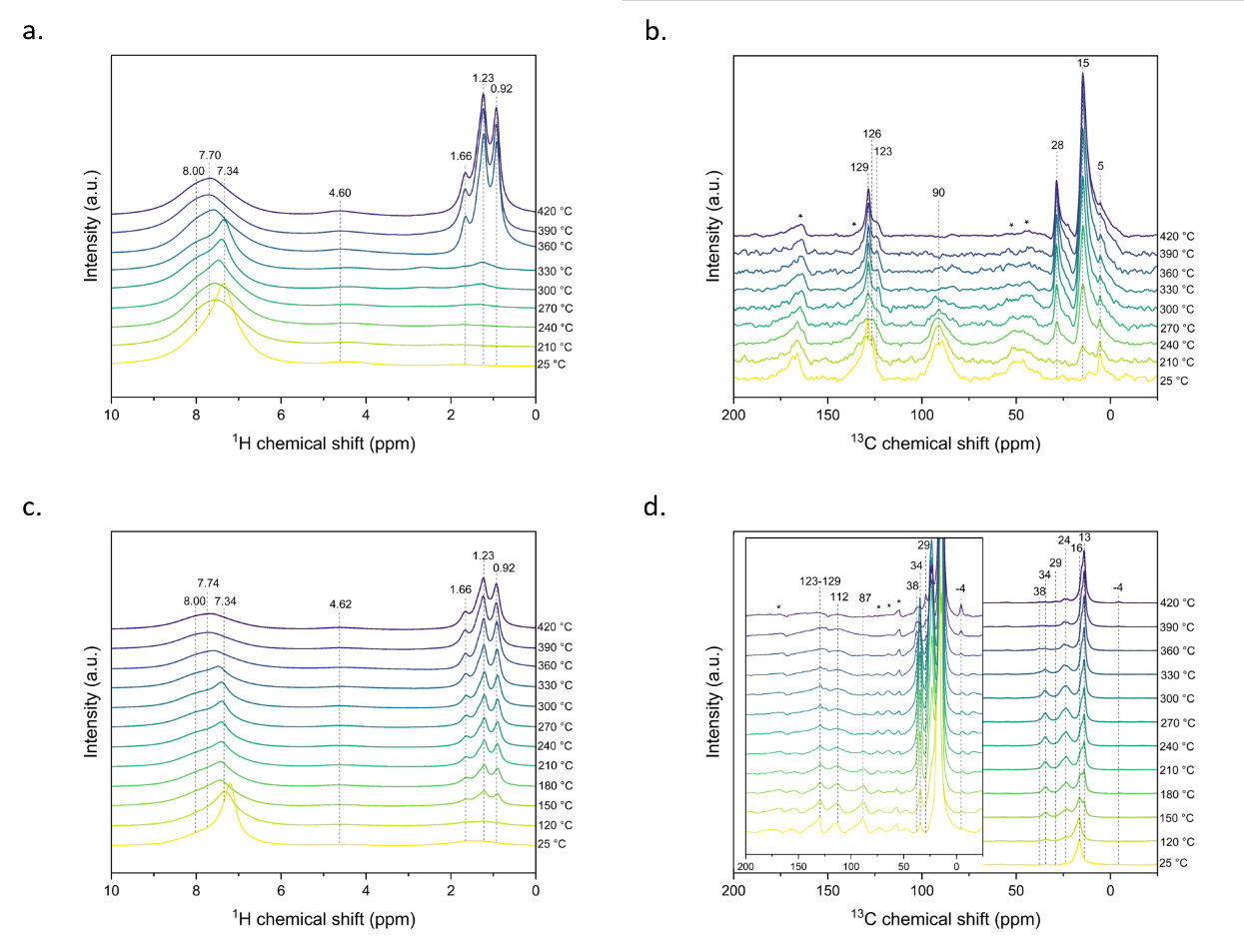


**Figure S9**: In situ a) ^1^H MAS NMR and b) ^13^C HPDEC spectra acquired during the reacting ethane-reacted Cu(I)-MOR with benzene at different temperatures. Similarly, c) and d) respectively represent in situ ^1^H MAS NMR and ^13^C HPDEC spectra acquired during the reacting propane-reacted Cu(I)-MOR with benzene at different temperatures. Asterisks are designating side bands, as attributed from Figure S6.


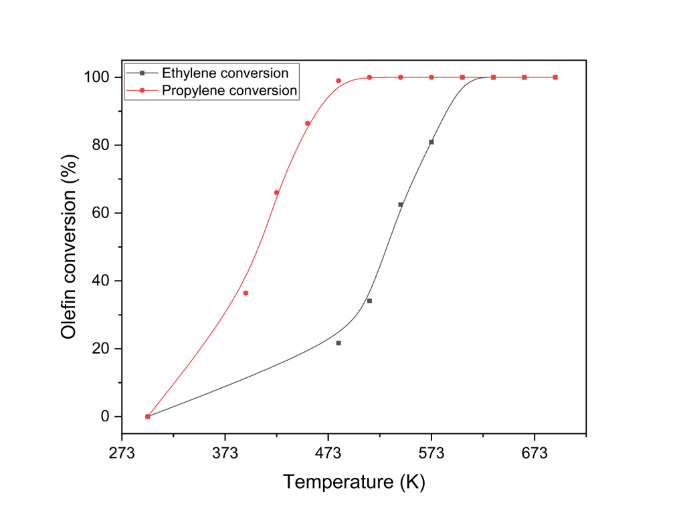


**Figure S10**: Conversion of ethylene and propylene π-bounded species during reaction with benzene on Cu(I)-MOR at different reaction temperature Tr, calculated based on HPDEC ^13^C MAS NMR signals. For π-bounded ethylene, the signal at 90 ppm (=CH_2_) was integrated from Figure 4a while for π-bounded propylene, the signal at 87 ppm (=CH_2_) was integrated from Figure 4c.


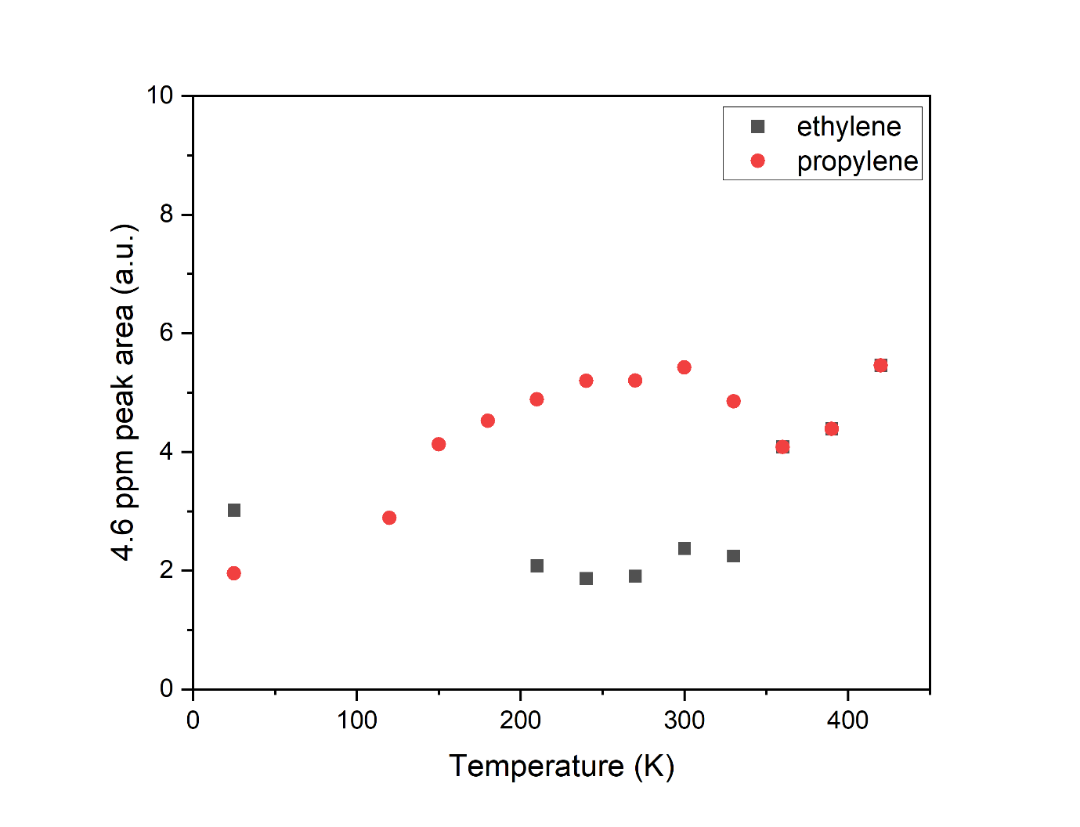


**Figure S11**: Evolution of the integral intensity of the ^1^H MAS NMR signal at around 4 ppm due to Brønsted acid sites for ethylene and propylene, taken from Figure S9.

**Supplementary note of mechanism investigation**

We first considered a direct Cu(I)-mediated pathway for benzene alkylation as shown schematically in Figure S12a. The associated free energy diagram is shown in Figure S12b, and the DFT-optimized geometries of the key reaction intermediates are summarized in Figure 5c-f. This mechanism starts with ethylene π-bonded to Cu(I), while benzene is physisorbed within the zeolite pore. The first step involves carbon-carbon bond formation between C6_Bz_ and C2_Et_ that is followed by a hydrogen transfer from C6_Bz_ to C1_Et_. This step has a free energy barrier of ~ 1.8 eV at 298 K that increases to 2.0 eV at 623 K. The transition state for this C-C bond formation step involves partial detachment of the Cu(I) cation; Figure S12e shows that Cu remains bound to only one framework oxygen. The second hydrogen transfer step (i.e., of H6_Bz_) is significantly downhill and is followed by the formation of the alkylated product. As our experiments show that ethylbenzene forms at temperatures lower than 623 K and given the relatively high barrier for the direct mechanism, we conclude that the observed activity must arise due to a different reaction pathway.


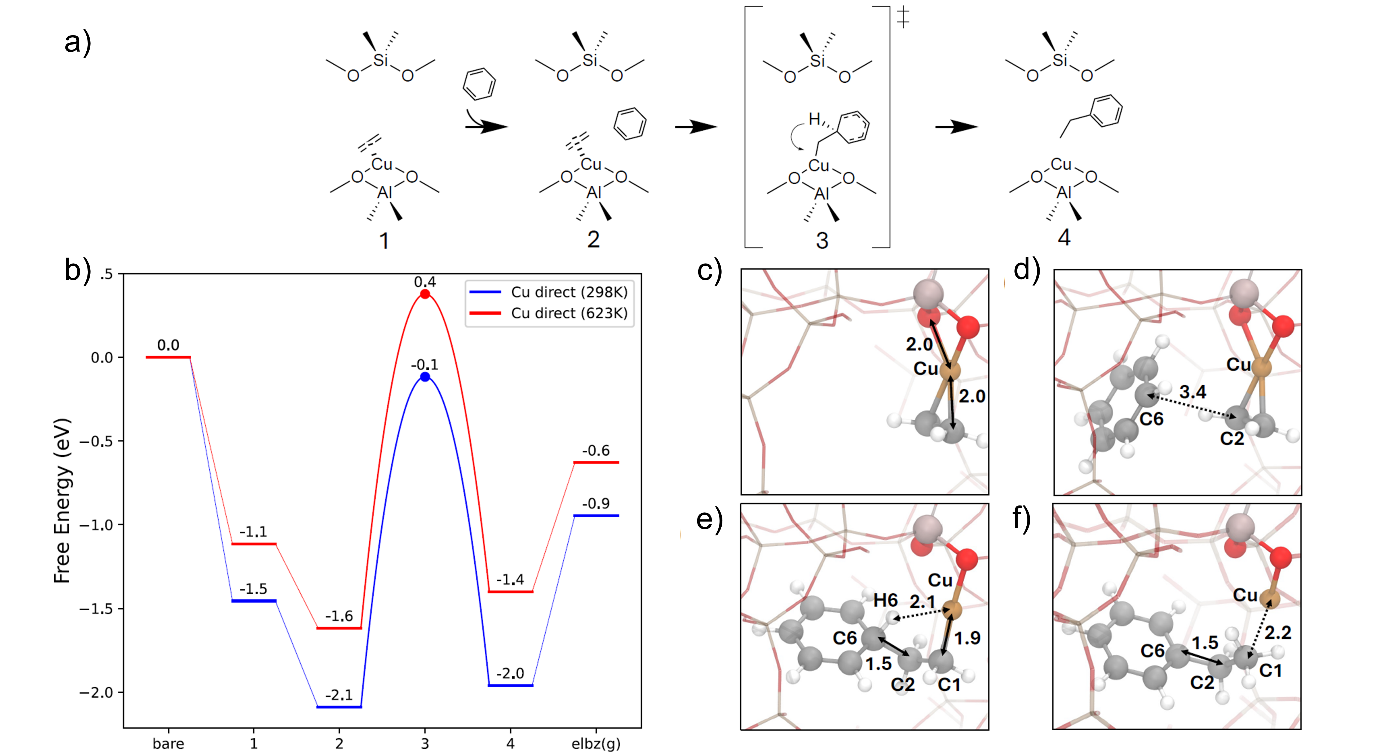


**Figure S12**: a) A proposed reaction scheme for benzene alkylation mediated by a Cu(I) site. b) Corresponding free energy profile calculated at 298 K (blue) and 623 K (red). c–f) Optimized structures of the key intermediates and transition state along the pathway, corresponding to states 1–4 in panel a). Color scheme: C (grey), H (white), O (red), Cu (brown), Al (pink). Numbers correspond to distance in Å.


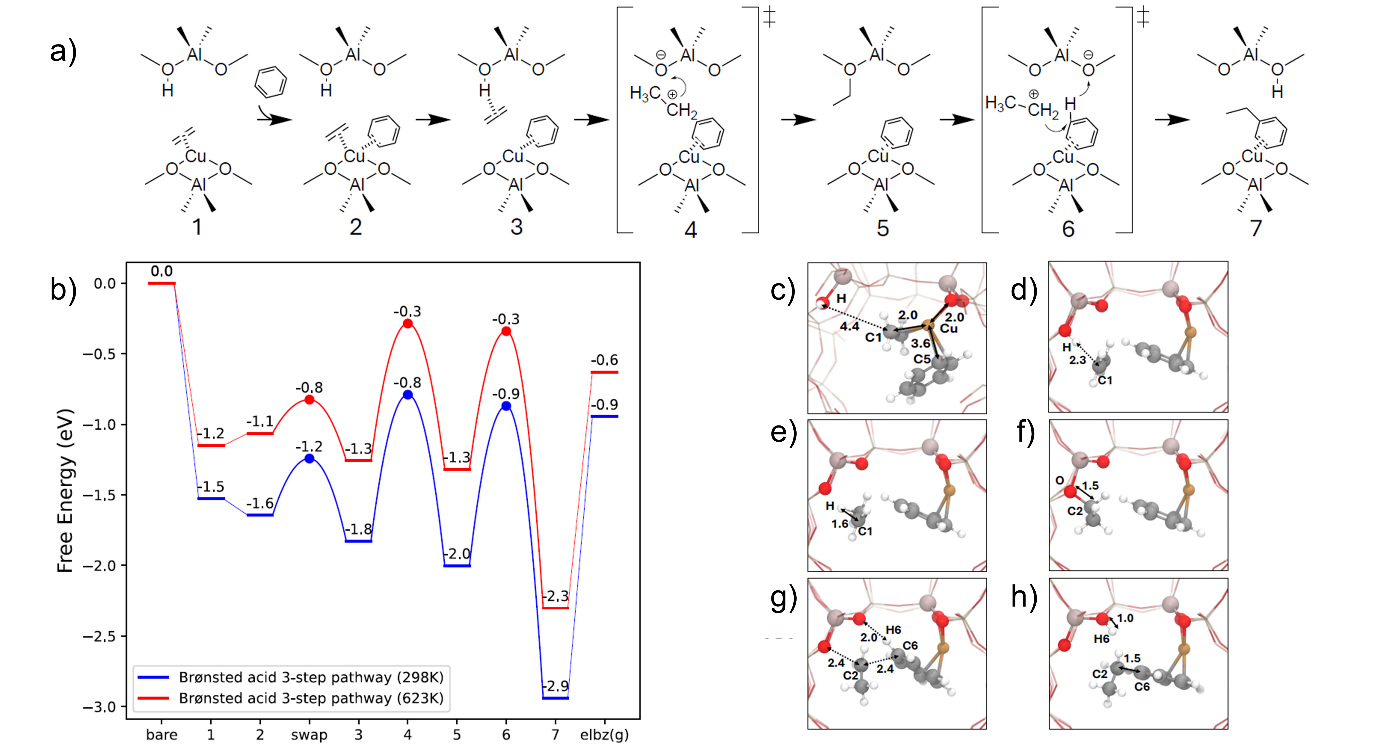


**Figure S13**: a) Proposed 3-step reaction scheme for benzene alkylation reaction mediated by the BH/Cu site. b) Corresponding free energy profile calculated at 298 K (blue) and 623 K (red). c–h) Optimized structures of the key intermediates and transition state along the pathway, corresponding to states 2–7 in panel a). Color scheme: C (grey), H (white), O (red), Cu (brown), Al (pink). Numbers correspond to distance in Å.

Next, we considered a pathway over bi-functional active site consisting of a Brønsted acid site (BH) located near the Cu(I) cation. For this bi-functional site, we first discuss a 3-step mechanism (Figure S13a) that includes: ethylene desorption (1 – 3), formation of framework-bound ethyl intermediate on BH (4 – 5), and C-C bond formation to yield ethylbenzene (5 – 7). We first investigated if the relatively high partial pressure of benzene could facilitate the desorption of Cu(I)-bound ethylene. Figure S13b (step: 2-3) shows that the free energy barriers associated with this swapping step are small (i.e., 0.4 eV at 298 K, and 0.3 eV at 623 K) suggesting that ethylene desorption is facile. Note that BH is not involved in the transition state of swapping step but helps in stabilizing the desorbed ethylene (Figure S13d). This configuration serves as the starting point for the alkylation reaction between BH-bound ethylene and Cu-bound benzene.

Specifically, the second step involves proton transfer from the BH site to ethylene (Figure S13b; step: 3-5) resulting in the formation of a framework bound ethoxy (Figure S13f.) This step has a free energy barrier of 1 eV. The third step (Figure S13b; step: 5-7) is the C-C bond formation between C2_Et_ and C6_Bz_ and simultaneous transfer of the H6_Bz_ to regenerate the Brønsted acid site. We observe a 6-membered ring transition state (Figure S13f) involving both the hydrocarbons and the framework oxygen atoms with a free energy barrier of 1.1 eV and 1.0 eV at 298 and 623 K, respectively. Although the energetics of this mechanism are more favorable than the direct pathway outlined above, we note that our FTIR and NMR experiments do not show formation of framework bound ethoxy species (Figures 4a, S13e). Thus, we consider another mechanism that sidesteps the formation of a framework bound ethoxy intermediate.

**Computational Methods**

All periodic density functional theory (DFT) calculations were conducted using the Vienna Ab-initio Simulation Package (VASP) with the revised Perdew-Burke-Ernzerhof (RPBE) exchange-correlation functional.^[2, 3]^ A plane-wave cutoff energy of 400 eV was used. The Brillouin zone was primarily sampled at the Γ-point. Dispersion interactions were considered using the Grimme D3 method with Becke-Jonson damping.^[4]^ Convergence criteria were established at 0.03 eV/Å for force and 10⁻⁶ eV for the electronic self-consistency cycle. Structural optimization permitted the movement of all atoms within the computational cell. For vibrational frequency calculations, all framework atoms were constrained, except for the aluminum (Al) atom and its neighboring oxygen (O) atoms. Free energies include zero-point energy corrections and harmonic contributions to the entropy. Nudge elastic band (NEB) calculations used 7 intermediate images; these configurations were generated manually or through interpolation.^[5]^ Atomic Simulation Environment (ASE) was used for analysis.^[6]^ Visual Molecular Dynamics (VMD) was used for visualization.^[7]^

In line with our previous study, we employed an ensemble-based approach to investigate all possible Cu(I) locations.^[8]^ For each of the four unique T-sites in the MOR zeolite, we evaluated six Cu(I) configurations to determine their stability. Two most stable Cu(I) configurations or each T-site were selected for further analysis. These configurations were then assessed for steric hindrance by attempting to physically adsorbed ethylene. The T4 site configurations has the strongest ethylene adsorption energies, and were chosen for subsequent NEB calculations.


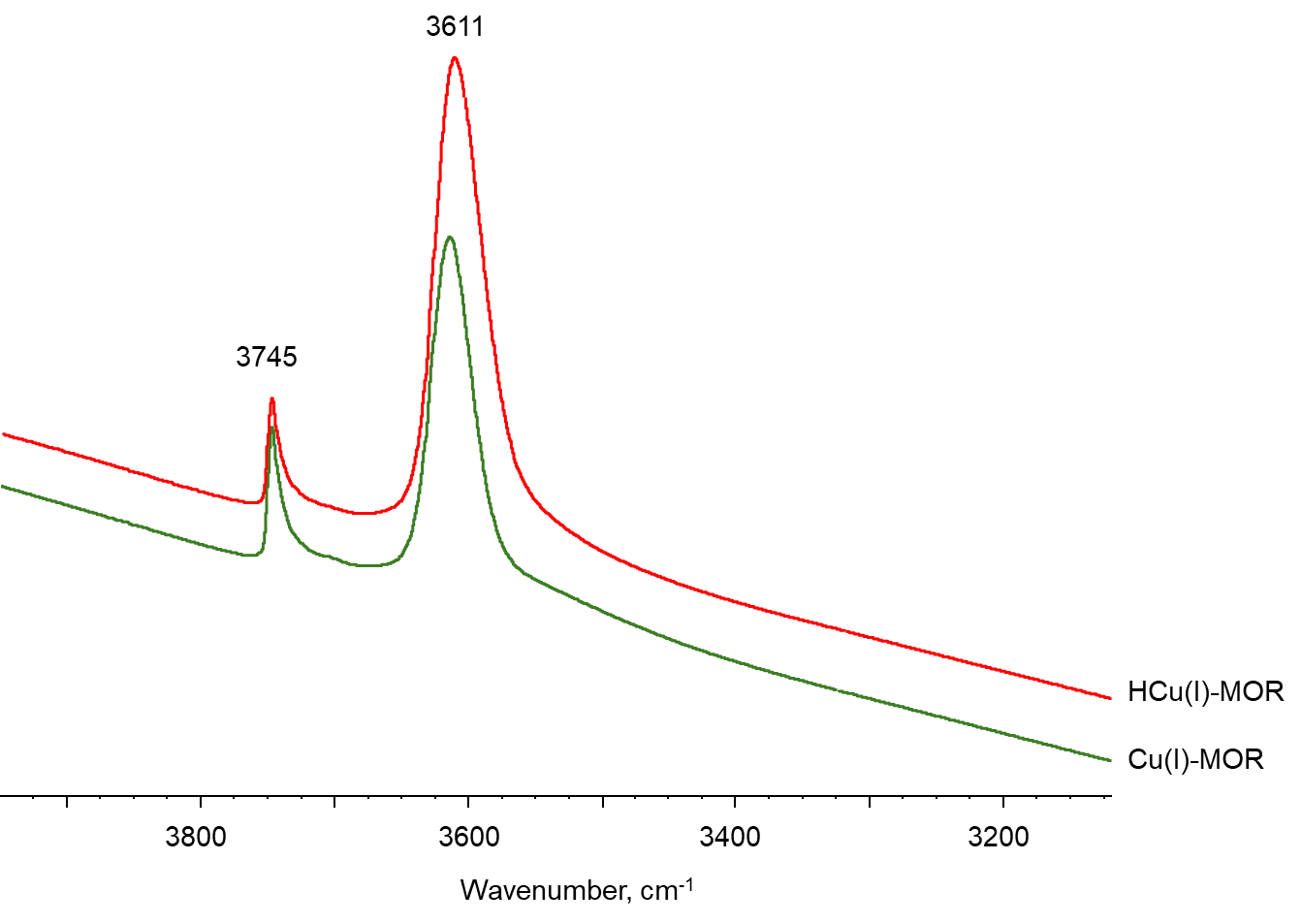


**Figure S14**: FTIR spectra of activated Cu(I)-MOR and HCu(I)-MOR, prepared using ammonium form of mordenite as starting material, aiming to increase the total number of Brønsted acid sites. The estimation of the number of BAS in HCu(I)-MOR was performed via integration of the band at 3611 cm^-1^ and comparing it to the number of BAS calculated for Cu(I)-MOR sample.


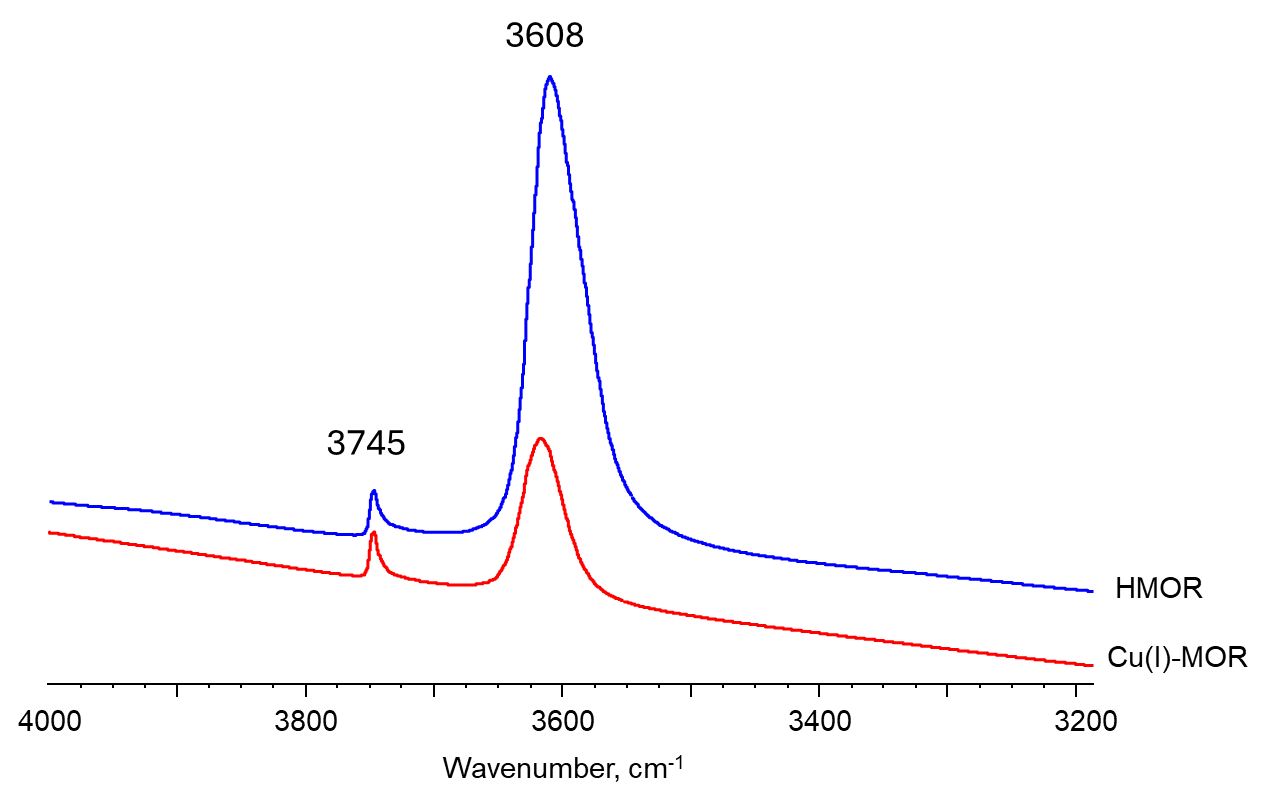


**Figure S15**: FTIR spectra of activated Cu(I)-MOR and HMOR prepared by in situ calcination of NH_4_MOR under vacuum at 723 K for 6 h.

**References**

1. Bennett, A. E.; Rienstra, C. M.; Auger, M.; Lakshmi, K. V.; Griffin, R. G. Heteronuclear Decoupling in Rotating Solids. *J Chem Phys* 1995, *103* (16), 6951–6958. <https://doi.org/10.1063/1.470372>.
2. Xing, S.-Y.; Zhang, H.; Liu, M.; Tang, Y.; Li, X.-H.; Zhao, Z.-J. First principles study on the alkylation of benzene with ethene over different zeolites: Insight into the intrinsic mechanism and structure-reactivity relationship. Mol. Catal. 2021, 512, 111762.
3. Hammer, B.; Hansen, L. B.; Nørskov, J. K. Phys. Rev. B 1999, 59, 7413–7421.
4. Grimme, S.; Antony, J.; Ehrlich, S.; Krieg, H. A consistent and accurate ab initio parametrization of density functional dispersion correction (DFT-D) for the 94 elements H–Pu. J. Chem. Phys. 2010, 132, 154104.
5. Jónsson, H.; Mills, G.; Jacobsen, K. W. *Nudged Elastic Band Method for Finding Minimum Energy Paths of Transitions.* In **Classical and Quantum Dynamics in Condensed Phase Simulations**; Berne, B. J., Ciccotti, G., Coker, D. F., Eds.; World Scientific: Singapore, 1998; pp 385–404.
6. Larsen, A. H.; Mortensen, J. J.; Blomqvist, J.; Castelli, I. E.; Christensen, R.; Dułak, M.; Friis, J.; Groves, M. N.; Hammer, B.; Hargus, C.; Hermes, E. D.; Mortensen, J. J.; et al. J. Phys.: Condens. Matter 2017, 29, 273002.
7. Humphrey, W.; Dalke, A.; Schulten, K. *VMD: Visual Molecular Dynamics.* *J. Mol. Graphics* **1996**, *14* (1), 33–38.
8. Guo, J.; Sours, T.; Holton, S.; Sun, C.; Kulkarni, A. R. Screening Cu‑Zeolites for Methane Activation Using Curriculum‑Based Training. ACS Catal. 2024, 14 (3), 1232–1242.
